# Antiprogestins in gynecological diseases

**DOI:** 10.1530/REP-14-0416

**Published:** 2015-01

**Authors:** Alicia A Goyeneche, Carlos M Telleria

**Affiliations:** Division of Basic Biomedical Sciences, Sanford School of Medicine, The University of South Dakota, Vermillion, South Dakota, 57069, USA

## Abstract

Antiprogestins constitute a group of compounds, developed since the early 1980s, that bind progesterone receptors with different affinities. The first clinical uses for antiprogestins were in reproductive medicine, e.g., menstrual regulation, emergency contraception, and termination of early pregnancies. These initial applications, however, belied the capacity for these compounds to interfere with cell growth. Within the context of gynecological diseases, antiprogestins can block the growth of and kill gynecological-related cancer cells, such as those originating in the breast, ovary, endometrium, and cervix. They can also interrupt the excessive growth of cells giving rise to benign gynecological diseases such as endometriosis and leiomyomata (uterine fibroids). In this article, we present a review of the literature providing support for the antigrowth activity that antiprogestins impose on cells in various gynecological diseases. We also provide a summary of the cellular and molecular mechanisms reported for these compounds that lead to cell growth inhibition and death. The preclinical knowledge gained during the past few years provides robust evidence to encourage the use of antiprogestins in order to alleviate the burden of gynecological diseases, either as monotherapies or as adjuvants of other therapies with the perspective of allowing for long-term treatments with tolerable side effects. The key to the clinical success of antiprogestins in this field probably lies in selecting those patients who will benefit from this therapy. This can be achieved by defining the genetic makeup required – within each particular gynecological disease – for attaining an objective response to antiprogestin-driven growth inhibition therapy.

**Free Spanish abstract:**

A Spanish translation of this abstract is freely available at http://www.reproduction-online.org/content/149/1/15/suppl/DC1.

## Introduction

Antiprogestins represent a family of compounds developed with the purpose of antagonizing the effect of progesterone on progesterone receptors (PR). Most derivatives are steroidal in nature and have mixed activities on the PR, ranging from pure antagonism to various degrees of agonistic effects contingent on the target tissue and the intracellular environment. Owing to these mixed activities on the PR, antiprogestins have been comprehensively categorized as PR modulators (PRMs). The degree of antagonistic or agonistic activity of the PRMs seems to depend on the balance among co-activators and co-repressors regulating the transcriptional activity of the PR, the intracellular molecular environment accounting for post-translational modifications, and the ratio of PR isoforms – i.e., PR-A vs PR-B, with PR-B having a strong transcriptional activation activity and PR-A being mostly transcriptionally inactive (Chabbert-Buffet *et al*. 2005, Hagan *et al*. 2012, Hagan & Lange 2014, Knutson & Lange 2014).

Paradoxically, the first compound with antiprogestin activity, originally termed RU-38486, was introduced to the scientific community in 1981 as a potent antiglucocorticoid agent (Philibert *et al*. 1981). RU-38486, or mifepristone, is a derivative of 19-nortestosterone with a dimethylaminophenyl moiety in position C11 that confers antagonistic properties. Its synthesis was part of an effort to develop an efficient antagonist against the glucocorticoid receptors (GR), which could be used to alleviate the consequences of excess glucocorticoid activity in patients with hypercortisolism (Baulieu 1997). During preclinical studies, it was rapidly discovered that mifepristone caused termination of pregnancy (Spitz & Bardin 1993, Baulieu 1997). This outcome was attributed to the fact that the compound was equally potent in antagonizing PR and GR (Cadepond *et al*. 1997). The lack of discrimination by mifepristone between PR and GR was not surprising considering the similarities between the structures of both steroid hormone receptors (Baulieu 1991). Additionally, it has been shown that mifepristone can also bind androgen receptors (ARs; Song *et al*. 2004), and further increase the complexity of the intracellular biochemistry of mifepristone; it has been recently shown that the steroid binds not only to the ligand-binding domain favoring repressor interaction and hindering receptor transactivation, but also to a second site representing the coactivator-binding domain of the ancestral 3-ketosteroid receptor, which is the ancestor of PR, GR, AR, as well as the mineralocorticoid receptor (Colucci & Ortlund 2013).

A large volume of studies explored the effect of mifepristone on different aspects of the mammalian reproductive axis. The contraceptive potential of mifepristone was extensively assessed in terms of its capacity to prevent ovulation, block implantation of a fertilized egg, and terminate early pregnancies (reviewed in Spitz *et al*. (1996)). The first human trial conducted with mifepristone in women with up to 8 weeks of amenorrhea led to termination of pregnancy in 80% of the cases (Herrmann *et al*. 1982). Shortly thereafter, it was shown that the efficacy of mifepristone abrogating early pregnancies by blocking PR was significantly enhanced when combined with a prostaglandin analog that potentiates uterine contractions (Bygdeman & Swahn 1985). Thus, the combination of mifepristone with a prostaglandin analog was adopted in many countries for medical termination of pregnancies at the first trimester (Spitz & Bardin 1993). In the USA, the combination of mifepristone and misoprostol was approved in 2000 for interrupting gestations of up to 49 days since the last menstrual period (Ellertson & Waldman 2001).

The antiglucocorticoid effect of mifepristone has been amply documented (reviewed in Agarwal (1996)), with the main application being the mitigation of the clinical manifestations of endogenous hypercortisolism (Nieman *et al*. 1985). After a successful multicenter trial (Fleseriu *et al*. 2012), mifepristone was approved in 2012 by the US Food and Drug Administration (http://www.fda.gov/newsevents/newsroom/pressannouncements/ucm292462.htm) to control hyperglycemia in patients with endogenous Cushing's syndrome associated with type 2 diabetes. As mifepristone does not differentiate between PR and GR, a series of efforts were conducted to develop compounds capable of modulating either receptor without impinging on the other. The group of PRMs with a potent antiprogesterone activity and a minimal antiglucocorticoid effect includes ZK-98299 (onapristone), ZK-230211 (lonaprisan), CDB-2914 (ulipristal), CDB-4124 (telapristone), ORG-31710, ORG-33628, and J-867 (asoprisnil) (reviewed in Chabbert-Buffet *et al*. (2005), Spitz (2006), and Lanari *et al*. (2012)), and, more recently, a 17-fluorinated steroid termed EC304 (Nickisch *et al*. 2013). Conversely, compounds with more antiglucocorticoid properties than antiprogesterone properties are also under development. They are termed GR modulators, are steroidal or non-steroidal in nature (Ray *et al*. 2007, Clark 2008, Belanoff *et al*. 2010, De Bosscher 2010, Gross *et al*. 2010), and are geared toward treating Cushing's syndrome as well as other conditions in which excess GR activity needs to be depressed without affecting PR-mediated physiological processes. Examples of such conditions are psychotic depression (reviewed in Benagiano *et al*. (2008)), weight gain (Belanoff *et al*. 2010), and glaucoma associated with high-dose glucocorticoid therapy (reviewed in Kersey & Broadway (2006)).

Herein, we review the current evidence supporting the efficacy of antiprogestins in attenuating the proliferation of cells encompassing a spectrum of benign and malignant gynecological diseases characterized by excessive cell division. We further describe the molecular mechanisms demonstrated and proposed that explain the growth-inhibitory properties of antiprogestins in cells of different genetic backgrounds and tissues of origin. The antiproliferative properties of antiprogestins are not always justified by a mere blockage by the compounds of the transactivation activity of the PR. Consequently, the term ‘antiprogestins,’ when stating the antigrowth activity of the compounds, is misleading. Yet, we have used the term to permit the allocation, under a unique chemical framework, of all compounds mentioned in this article. Timely, when the mechanisms driving the antigrowth effect of these synthetic steroid derivatives become widely understood, a better descriptor to represent this family of compounds will certainly arise.

## Breast cancer

Breast cancer is second only to lung cancer as the most common cause of cancer-related death in women (Siegel *et al*. 2013). Estrogen and progesterone have both been involved in breast carcinogenesis and progression (reviewed in Knutson & Lange (2014)). While antiestrogen therapy reached clinical application with the use of blockers of estrogen receptors (ER; e.g., tamoxifen and fulvestrant) or inhibitors of estradiol (E_2_) synthesis known as aromatase inhibitors (exemestane, anastrozole, and letrozole) (reviewed in Journe *et al*. (2008)), the antagonism of progesterone activity has not yet reached clinical practice. Nevertheless, evidence accumulated over the past 20 years suggests that antiprogesterone therapy for breast cancer has a large potential to be soon included within the armamentarium of approaches to treat breast cancer (reviewed in Horwitz (1992), Lanari *et al*. (2012), Giulianelli *et al*. (2013), Knutson & Lange (2014), and Muti (2014)). For instance, as monotherapy, mifepristone was demonstrated to block the proliferation of breast cancer cells carrying PR and ER, such as T-47D and MCF-7 cells in the absence or presence of estrogens (Bardon *et al*. 1985, Horwitz 1985, Gill *et al*. 1987, Musgrove *et al*. 1997). However, because mifepristone also blocked the proliferation of ER-negative/PR-negative MDA-MB-231 breast cancer cells (Liang *et al*. 2003), the relevance of PR as a mediator of the antiproliferative action of mifepristone in breast cancer cells is controversial. Contrasting this large volume of evidence for an inhibitory effect of mifepristone on breast cancer cell growth, growth stimulation by mifepristone in T-47D and MCF-7 cells was also timely reported (Bowden *et al*. 1989, Jeng *et al*. 1993), suggesting that the concentration of mifepristone used, and the presence or absence of progesterone and E_2_ in the culture media to name some variables, may justify such different outcomes in otherwise similar cell lines.

Mifepristone also had an additive interaction leading to the death of MCF-7 cancer cells when coupled with antiestrogens such as tamoxifen (El Etreby *et al*. 1998) or 4-hydroxytamoxifen (Schoenlein *et al*. 2007); it also inhibited the growth of and killed MCF-7 cells in combination with the Chk1 inhibitor, 7-hydroxystaurosporine (Yokoyama *et al*. 2000), and the aromatase inhibitor anastrozole (Gil *et al*. 2013). Mifepristone also interrupted the proliferation of MCF-7 sublines that were made resistant to 4-hydroxytamoxifen (Gaddy *et al*. 2004). More recently, mifepristone pretreatment has been reported to potentiate the toxicity of paclitaxel in mammary fat pad xenografts of ER-negative/PR-negative/GR-positive MDA-MB-231 cells (Skor *et al*. 2013).

Growth inhibition of PR-positive T-47D cells was reported with other antiprogestins, such as onapristone (Classen *et al*. 1993), ORG-31710 (Musgrove *et al*. 1997), lonaprisan (Afhuppe *et al*. 2010, Busia *et al*. 2011), telapristone (Gupta *et al*. 2013*a*), and EC304 (Nickisch *et al*. 2013).

Valuable information on the anti-breast-cancer effect of antiprogestins was generated from mouse models of breast cancer. For instance, mice genetically engineered to lack expression of p53 and BRCA1 tumor suppressors in their mammary glands develop spontaneous aggressive PR-overexpressing breast tumors, which can be prevented by the administration of mifepristone (Poole *et al*. 2006). Breast tumors induced by continuous administration of medroxyprogesterone acetate (MPA) in BALB/c mice and expressing a high PR-A to PR-B ratio respond to antiprogestins mifepristone, onapristone, or lonaprisan with inhibition of tumor growth and apoptosis (Montecchia *et al*. 1999, Helguero *et al*. 2003, Simian *et al*. 2006, Wargon *et al*. 2009). The response of these tumors to mifepristone is associated with an increase in tumor stroma and microvasculature, which allows better access to other chemotherapeutic agents such as paclitaxel or doxorubicin when provided in nanoparticle formulations, leading to a better therapeutic outcome (i.e., further growth inhibition than that caused by paclitaxel or doxorubicin alone) (Sequeira *et al*. 2014). In mammary gland tumors induced in rats by 7,12-dimethylbenzanthracene (DMBA), concomitant administration of mifepristone significantly delayed tumor development (Bakker *et al*. 1987), whereas administration of mifepristone to animals with pre-established DMBA-induced tumors abrogated tumor progression. This latter effect was additive to tamoxifen, leading to further growth inhibition (Bakker *et al*. 1989). DMBA-induced mammary tumors in rats were also significantly inhibited by onapristone, ulipristal, and ORG-31710 (Michna *et al*. 1989, Kloosterboer *et al*. 2000, Wiehle *et al*. 2007).

In patients with breast cancer, clinical trials with antiprogestins have had only partial responses. In one study, 200 mg mifepristone given daily for 3 months in tamoxifen-resistant breast tumors generated a positive response in 18% of patients (Romieu *et al*. 1987). In another study in patients with metastatic breast cancer resistant to tamoxifen, daily doses of 200–400 mg mifepristone led to an objective response in seven out of 11 patients (Klijn *et al*. 1989), whereas in another study with 28 patients treated with daily doses of 200 mg mifepristone, only three patients showed a response (Perrault *et al*. 1996). In a clinical trial using onapristone, 75% of patients responded to the treatment with an objective response; yet, the compound prompted liver toxicity, which discouraged further use (Robertson *et al*. 1999). A recent clinical trial with lonaprisan has reported limited efficacy in advanced stage IV, PR-positive, HER2-negative, metastatic breast cancer (Jonat *et al*. 2013). In summary, the apparent lack of robust objective responses reported in patients with breast cancer when receiving antiprogestins has been attributed to the lack of patient stratification according to their molecular profile, in particular the proportion of expression of PR-A relative to PR-B (Lanari *et al*. 2012, Giulianelli *et al*. 2013), and the lack of assessment of PR target gene signatures in responders vs non-responders (Hagan & Lange 2014, Knutson & Lange 2014). As breast cancer comprises four major subtypes with specific molecular drivers and histological characteristics (Cancer Genome Atlas Network 2012), new clinical trials should be tailored only to select groups of patients whose tumor genetics and PR target gene signatures make them suitable candidates to likely benefit from antiprogesterone therapy.

In terms of breast cancer prevention, antiprogestin therapy also has potential application. For instance, when antiprogestin mifepristone was provided to premenopausal women before a scheduled hysterectomy as a consequence of a leiomyoma, needle biopsies from mammary tissue clearly demonstrated a reduction in epithelial cell proliferation, suggesting the chemopreventive nature of the drug (Engman *et al*. 2008).

## Ovarian cancer

Ovarian cancer is the most lethal disease of the female reproductive track. Its 5-year survival below 50% has not changed for the past 30 years, indicating the need for new therapeutic interventions (reviewed in Vaughan *et al*. (2011), Modugno *et al*. (2012), Romero & Bast (2012), and Coleman *et al*. (2013)). The first reported effect of antiprogestins in ovarian cancer was in 1996 when it was demonstrated that mifepristone efficiently blocked the growth of A2780 and OVCAR-3 cells *in vitro* (Rose & Barnea 1996). Thereafter, it was reported that mifepristone potentiated the toxicity of cisplatin against COC1 ovarian cancer cells (Qin & Wang 2002, Li *et al*. 2003), and that onapristone and mifepristone inhibited cell growth and the synthesis of DNA in ML5 and ML10 human ovarian cystadenoma cells, as well as in HOC-7 and OVCAR-3 ovarian cancer cells (Zhou *et al*. 2002). The OVCAR-3 cell growth block by mifepristone was further confirmed in 2006 (Fauvet *et al*. 2006). Using various ovarian cancer cell lines of different genetic backgrounds, our laboratory additionally found that mifepristone blocked cell growth *in vitro* and demonstrated its efficacy *in vivo* at doses of 0.5 or 1 mg/day in mice carrying ovarian cancer xenografts (Goyeneche *et al*. 2007). We observed that when used at concentrations likely achievable in the clinic (i.e., pharmacological), mifepristone, ulipristal, and ORG-31710 all had cytostatic effects, with cells returning to the cell cycle upon drug removal; however, if used at suprapharmacological doses, the antiprogestins, instead, killed the cells (Goyeneche *et al*. 2007, 2012). We further reported that adding antiprogestin mifepristone following a platinum agent potentiates platinum lethality and improves overall treatment efficacy (Freeburg *et al*. 2009*a*), and that resistance to platinum and/or paclitaxel does not affect the sensitivity of ovarian cancer cells to antiprogestin-mediated cytotoxicity (Freeburg *et al*. 2009*b*, Gamarra-Luques *et al*. 2014). The repopulation of ovarian cancer cells that escaped platinum or platinum/paclitaxel therapy was also blocked by chronic presence of antiprogestin mifepristone (Freeburg *et al*. 2009*a*, Gamarra-Luques *et al*. 2012), providing evidence for a long-term use of antiprogestins as antirepopulation therapy from a minority of cells that escaped otherwise effective chemotherapies (Telleria 2013).

Despite the evidence of antiprogestins being efficient in blocking ovarian cancer cell growth, clinical studies on the subject have been very limited. In 2000, in a phase II clinical trial, 34 patients with recurrent ovarian cancer no longer responsive to cisplatin–paclitaxel chemotherapy were treated daily with 200 mg oral mifepristone in courses of 28 days. Nine patients had a response to mifepristone showing a decrease in tumor size by at least 50%, or a 50% decline in antigen CA-125 used to assess disease recurrence (Rocereto *et al*. 2000). However, a second phase II clinical trial including 24 patients with advanced ovarian cancer that recurred from standard chemotherapy within 6 months showed that only one patient had an objective response to a 28-day course of 200 mg mifepristone given daily (Rocereto *et al*. 2010). This clinical evidence is highly limited in terms of the number of patients, the lack of predictive biomarkers of response, and the fact that the studies consider ovarian cancer as a single disease.

There is growing consensus that ovarian cancer is a very heterogeneous disease, not only from the histological standpoint, but also genetically and within their different histological subtypes (Cancer Genome Atlas Research Network 2011, Vaughan *et al*. 2011). For instance, the value of patient stratification by disease subtype has been recently evidenced in a study by the Ovarian Tumor Tissue Analysis (OTTA) consortium including almost 3000 women worldwide with invasive epithelial ovarian cancer (Sieh *et al*. 2013). The study provided a significant positive association between expression of PR and survival advantage in patients with high-grade serous and endometrial ovarian cancers, but not in patients with mucinous, clear cell, or low-grade serous ovarian carcinomas. Then, it became clear that new clinical studies should be conducted stratifying patients by disease subtype and PR expression status, with the overall goal of defining the genetic backgrounds of the ovarian cancers that would likely respond to antiprogestin therapy. Moreover, clinical studies using antiprogestins other than mifepristone are required. Finally, the dynamic of expression of PR in the ovarian cancer cells and the cells encompassing the accompanying tumor stroma is crucially needed in order to define whether direct or paracrine cancer–stroma interactions explain the antiproliferative actions of antiprogestins.

## Endometrial cancer

Endometrial carcinoma is a frequent malignancy of the female reproductive track (reviewed in SGO Clinical Practice Endometrial Cancer Working Group *et al*. (2014*a,b*)). Information on the putative therapeutic benefit of antiprogestins for these patients, however, is scarce. Mifepristone was shown to bind estrogen-independent PR in the human endometrial cell line IK-90 and prevent the growth inhibition induced by the synthetic progestin R5020 (Terakawa *et al*. 1988). By contrast, mifepristone blocked growth and promoted cell death in EM-42 endometrial cells established from a benign endometrium (Han & Sidell 2003). Similarly, in the well-differentiated human endometrial adenocarcinoma Ishikawa cells, which express a functional PR (Lessey *et al*. 1996), mifepristone blocked their growth (Li *et al*. 2005, Navo *et al*. 2008, Moe *et al*. 2009). Clinically achievable doses of mifepristone also inhibited the growth of three endometrial cancer cell lines (Hec-1A, LEK, and RL95-2), while inducing a decline in the abundance of GR (Schneider *et al*. 1998).

## Cervical cancer

Cervical cancer is highly frequent worldwide and its development almost always is associated with previous infection with human papillomavirus (HPV; reviewed in Haie-Meder *et al*. (2010), and Meijer & Snijders (2014)). In the cervical adenocarcinoma cell line C4-1, mifepristone sensitized the cells to the toxicity of gamma irradiation by reversing dexamethasone-induced *HPV E6/E7* mRNA expression, p53 inhibition, and survival effects, all opposing the deleterious effects of radiation therapy (Kamradt *et al*. 2000). Mifepristone also blocked the growth of HeLa cervical adenocarcinoma cells *in vitro* and *in vivo*, synergizing with cisplatin-induced toxicity (Jurado *et al*. 2009). More recently, a reversal of the resistance to mitomycin-C by mifepristone has been described in HeLa cells (Chen *et al*. 2014).

## Leiomyoma

Uterine leiomyoma, also known as uterine fibroid, represents a benign tumor of the smooth muscle cells of the myometrium that relies on estrogen and progesterone to grow (Murphy & Castellano 1994, Shimomura *et al*. 1998, Yoshida *et al*. 2010). The utilization of PRMs to interrupt leiomyoma growth has been quite successful. For instance, patients receiving 25 or 50 mg mifepristone daily for 3 months exhibited a decrease in the size of the leiomyomata by 50% after concluding the treatment and without displaying any significant side effect (Murphy *et al*. 1993, 1995). Mifepristone reduced growth and ameliorated the symptoms in premenopausal women with large leiomyomata (Eisinger *et al*. 2003). Women with leiomyoma, who were treated with low doses of mifepristone for 6 months, showed a significant reduction in their uterine volume, bleeding, and pain, which resulted in an increase in their overall quality of life (Eisinger *et al*. 2009). In another study, 30 women with uterine leiomyomata scheduled for surgery received 50 mg mifepristone for 3 months before the operation and showed a significant reduction in the leiomyoma volume concurrently with reduced bleeding (Engman *et al*. 2009). A meta-analysis of 11 clinical trials performed using treatment with mifepristone ranging from 2.5 to 25 mg/day for 3–6 months concluded that the antiprogestin was globally effective, resulting in the decline of the volume of the uterus and the leiomyoma, as well as the alleviation of leiomyoma symptoms, including blood loss, pelvic pain, and pressure, without any evidence of causing endometrial hyperplasia or atypia (Shen *et al*. 2013). Finally, a phase II clinical trial performed in women with symptomatic uterine leiomyomata reported that vaginal mifepristone given at a dose of 10 mg daily for 3 months was a safe and effective way of controlling bleeding and reducing the volume of the fibroids (Yerushalmi *et al*. 2014).

Ulipristal also was shown to be effective in reducing the number of viable primary cultured leiomyoma cells in a dose-dependent manner (Xu *et al*. 2005). The reduced antiglucocorticoid effect of ulipristal, when compared with mifepristone, might be beneficial for long-term treatment schedules. For instance, in a randomized controlled trial, administration of ulipristal for 3 months led to a reduction in the volume of the tumors and improved the quality of life without any serious side effects; these would have included the lack of antiglucocorticoid effects and uterine hyperplasia observed, sometimes, as a consequence of prolonged mifepristone activity that precludes estrogenic effects from being counteracted by progesterone (Levens *et al*. 2008). A clinical trial comparing ulipristal given at doses of either 5 or 10 mg daily to patients with symptomatic uterine fibroids before surgery against a once-a-month injection of the gonadotropin-releasing hormone (GnRH) agonist leuprolide acetate used as a standard of care demonstrated similarities with the approaches in controlling uterine bleeding, with the advantage that ulipristal-treated patients were less likely to have hot flashes, very commonly induced by GnRH agonists due to suppression of E_2_ (Donnez *et al*. 2012).

The antiproliferative effect of ulipristal on primary leiomyoma cells was also mimicked by asoprisnil (Chen *et al*. 2006). Both asoprisnil- and ulipristal-treated primary leiomyoma cells produced less extracellular matrix proteins, usually responsible for the fibrotic nature of the tumor, when compared with matched normal endometrial cells (Yoshida *et al*. 2010). A controlled clinical trial demonstrated that asoprisnil reduced the volume of leiomyoma, suppressed uterine bleeding, and improved patient's quality of life without causing hypoestrogenism (Chwalisz *et al*. 2007) and the consequent bone loss associated with other non-surgical treatment(s) that blocks the pituitary–ovarian axis (e.g., usage of GnRH agonists). Telapristone has also shown promise in inhibiting cell proliferation in primary cultures of uterine leiomyoma smooth muscle cells isolated from premenopausal women undergoing hysterectomy due to leiomyoma-associated symptomatology; this effect occurred without affecting the growth of control myometrial smooth muscle cells collected from adjacent corresponding uteri (Luo *et al*. 2010).

## Leiomyosarcoma

Leiomyosarcoma is a rare malignant tumor from smooth muscle cells most commonly originating in the uterus. Some sporadic cases of low-grade uterine leiomyosarcoma have been reported as responding to mifepristone therapy. For instance, daily administration of 200 mg mifepristone in a patient with low-grade leiomyosarcoma with osteolytic metastasis showed a 5-year regression of the bone tumors (Baulieu 1997). Another patient with PR-positive, low-grade leiomyosarcoma displayed a 3-year regression response to 50–200 mg of mifepristone daily (Koivisto-Korander *et al*. 2007).

## Endometriosis

Endometriosis is a condition characterized by the presence of endometrial glands and stroma outside the uterus. It is a common cause of infertility and affects up to 17% of women of reproductive age (reviewed in Giudice & Kao (2004), Hansen & Eyster (2006), and Bernardi & Pavone (2013)). In 1991, the efficacy of mifepristone on this disease was first explored by administering a dose of 100 mg/day for 3 months to six cycling women diagnosed with endometriosis. The treatment showed improved relief of pelvic pain in all subjects (Kettel *et al*. 1991). A study by the same group confirmed the finding in another cohort of subjects 3 years later (Kettel *et al*. 1994), and the overall data were timely reviewed (Murphy & Castellano 1994). Further studies using 50 mg/day mifepristone concluded that endometriosis regressed by half after 6 months of treatment (Kettel *et al*. 1996).

In a rat model of experimental endometriosis generated by implanting endometrium into the peritoneal cavity, animals receiving mifepristone for 8 weeks did not show any blockage of disease progression (Tjaden *et al*. 1993). Onapristone, on the other hand, when tested in another model of surgically induced endometriosis in intact rats reduced the growth of endometrioid foci by 40% without affecting the proliferation of the eutopic endometrium (Stoeckemann *et al*. 1995). More recent studies with mifepristone have shown that when the drug was administered in slow-release pellets to rats, it was able to slow the growth of endometrial explants used as a model of endometriosis *in vivo* (Mei *et al*. 2010). Another recent study using a rat model of surgically induced endometriosis has provided evidence that ulipristal, given as oral daily doses of 0.1 mg for 2 months, reduced the size of endometrioid foci by at least 50% and was associated with a decline in the number of cells showing expression of the proliferation marker Ki67 (Huniadi *et al*. 2013). Finally, in a study on monkeys with surgical induction of endometriosis, mifepristone caused thinning of the pelvic endometrioid lesions similar to that caused by GnRH agonists, yet with the benefit of not causing hypoestrogenism and the consequent bone loss (Grow *et al*. 1996).

## Mechanisms of growth inhibition driven by antiprogestins

The molecular mechanisms triggered by antiprogestin(s) while blocking cell growth are multiple and not yet fully understood. In the following section, we describe the molecules and pathways that either directly or indirectly have been reported to be involved in antiprogestin-induced cell growth inhibition. The section is not limited to the gynecological diseases described previously: it expands on mechanisms uncovered while antiprogestins block the growth of non-gynecological-related cancer cells including prostate (El Etreby *et al*. 2000, Liang *et al*. 2002), meningioma (Matsuda *et al*. 1994, Grunberg *et al*. 2006), glioblastoma (Pinski *et al*. 1993), osteosarcoma (Tieszen *et al*. 2011), and gastric adenocarcinoma (Li *et al*. 2004*a*).

### Antiprogestin-induced cell cycle arrest

A large number of studies support the concept that, when antiprogestins block cell growth, one key mechanism involved is the arrest of the cell cycle. Such arrest mostly occurs in the G1 phase and involves upregulation of cyclin-dependent kinase (Cdk) inhibitors p21^cip1^ and/or p27^kip1^ and their re-localization to the nuclear compartment and inhibition of the G1/S kinase Cdk2, thus not allowing DNA synthesis and cell division to proceed further.

The tumor-promoting capacity of progesterone, the role of PR, and the effect of antiprogestins on mammary tumor development were in part drawn from a series of studies using a mouse model in which chronic exposure of female BALB/c mice to MPA induces ductal mammary adenocarcinoma (reviewed in Lanari *et al*. (2009)). *In vivo*, in these MPA-induced tumors, daily treatment with mifepristone or onapristone led to tumor growth retardation in association with increased expression of p21^cip1^ (Peters *et al*. 2001). Furthermore, lung and axillary metastases caused by the MPA-induced tumor line termed C7-2-HI, which expresses high levels of PR and ER, underwent complete regression when the animals received estrogens in combination with mifepristone; a reduction in metastatic growth was associated with increased expression of p21^cip1^ and p27^kip1^ (Vanzulli *et al*. 2005). *In vitro*, in two metastatic cell lines originated from MPA-induced mouse ductal mammary adenocarcinomas, mifepristone or onapristone reduced the number of mitosis and cell growth, while increasing expression of p21^cip1^, p27^kip1^, and p53 (Vanzulli *et al*. 2002). Concordant with these data from mice, in DMBA-induced experimental mammary tumors in rats, treatment with antiprogestins induced accumulation of cells in the G0/G1 phase of the cell cycle with a concomitant reduction in cells transiting the S and G2/M phases (Michna *et al*. 1992). This outcome is consistent with a phenotype of differentiation as the number of mitotically active cells is reduced in association with a cell phenotype resembling that of non-proliferative secretory cells (Michna *et al*. 1989).

In human MCF-7 breast cancer cells, mifepristone showed synergistic cytotoxicity with 4-hydroxytamoxifen in association with downregulation of retinoblastoma (Rb) tumor suppressor (Schoenlein *et al*. 2007). When treated only with mifepristone, MCF-7 cells were arrested at the G1 phase of the cell cycle (Fjelldal *et al*. 2010). In T-47D and BT-474 breast cancer cells, mifepristone and ORG-31710 reduced the number of cells transiting the S phase and increased the abundance of hypo-phosphorylated (inactive) Rb, thus arresting the cells at the G1 phase of the cell cycle in association with an increase in p21^cip1^ expression and reducing cyclin E/Cdk associated kinase activity (presumably Cdk2) (Musgrove *et al*. 1997). Lonaprisan, on the other hand, also halted proliferation of T-47D breast cancer cells blocking the S phase entry induced by E_2_ (Afhuppe *et al*. 2010), inducing G1 cell cycle arrest, which required the upregulation of p21^cip1^ triggered by lonaprisan-bound PR to the p21^cip1^ promoter (Busia *et al*. 2011).

Endometrial Ishikawa cancer cells treated with mifepristone underwent cell cycle arrest with a decline in the proportion of cells transiting G2/M and an increase in cells in the S phase (Li *et al*. 2005); yet, other reports with the same cells indicate that cell cycle arrest occurred in G1 in a manner likely depending on the induction of p53 (Navo *et al*. 2008, Moe *et al*. 2009). Cell cycle arrest by mifepristone involving upregulation of p21^cip1^ was also shown in Hec-1A endometrial cancer cells (Schneider *et al*. 1998).

In ovarian cancer cells, mifepristone used at concentrations likely to be achieved *in vivo* induced G1 cell cycle arrest and inhibition of synthesis of DNA as measured by BrdU incorporation (Goyeneche *et al*. 2007). In agreement, inhibition of DNA synthesis as measured by ^3^H-thymidine incorporation was observed after mifepristone treatment in cultured macrophages (Roberts *et al*. 1995). We also observed that mifepristone-treated cells had low expression of E2F transcription factor required for S phase transit and reduced activity of Cdk2 required for Rb hyper-phosphorylation and consequent activation (Goyeneche *et al*. 2007). A decline in Cdk2 activity by ORG-31710 associated with increased p21^cip1^ was also reported in T-47D breast cancer cells (Musgrove *et al*. 1997).

Cdk2 activity is necessary to promote S phase entry (Conradie *et al*. 2010). As such, Cdk2 triggers the transition in the cell cycle from G1 by stimulating histone gene transcription (Zhao *et al*. 2000). To be active and available, Cdk2 should bind to cyclin E, be allocated in the nuclear compartment, and not be bound to the Cdk inhibitors p21^cip1^ and p27^kip1^ (Lents *et al*. 2002, Brown *et al*. 2004, Conradie *et al*. 2010). Thus, by promoting p21^cip1^/p27^kip1^ upregulation and favoring their nuclear localization, antiprogestins promote the decline in Cdk2 nuclear activity and, consequently, the progression of the cell cycle. We have shown in ovarian cancer cells that mifepristone, ORG-31710, ulipristal, telapristone, 17α-hydroxy CDB-4124, and CDB-4453 (a demethylated derivative of CDB-4124) all increase p21^cip1^ and p27^kip1^ (Goyeneche *et al*. 2012, Gamarra-Luques *et al*. 2014). We have also established that, with a potency of mifepristone>ORG-31710>ulipristal, these antiprogestins increased p21^cip1^ and p27^kip1^ levels in the nuclear compartment while reducing cyclin E levels, consequently leading to an abrupt reduction in the nuclear activity of Cdk2 (Goyeneche *et al*. 2007, 2012). Reduction in the activity of Cdk2 by antiprogestins is relevant from a therapeutic standpoint because Cdk2 is often upregulated in ovarian cancer cells (Sui *et al*. 2001) and has been shown to be a valuable targetable molecule in ovarian (Etemadmoghadam *et al*. 2013) and breast (Achille *et al*. 2012), as well as other human cancer cells (Molenaar *et al*. 2009, Long *et al*. 2010). It is feasible that antiprogestins contribute to the recalibration of the activity of Cdk2 to that of normal cells.

Another cell fate phenotype that was reported to be associated with cell cycle arrest induced by antiprogestins is cellular senescence. Cellular senescence is a cell fate program described as permanent cell cycle arrest but with a very active and unique secretion phenotype termed senescence-associated secretory phenotype (SASP; reviewed in Perez-Mancera *et al*. (2014)). We have shown in LNCaP prostate cancer cells that exposure to mifepristone for 3 days caused a permanent cell cycle arrest that was not reversible upon the removal of the drug, yet was not associated with cell death. Instead, cells remained alive but irreversibly arrested, expressing the senescence marker, senescence-associated beta-galactosidase (SA-β-gal). This phenotype was not evident, however, in other cancer cells treated similarly, such as SKOV-3 (ovarian), U87MG (glioblastoma), or MDA-MB-231 (breast) (Brandhagen *et al*. 2013), suggesting that the senescence program requires a particular genetic underpinning. LNCaP cells, for instance, but not the other cells studied, express the tumor suppressor CDKN2A (p16^INK4^), which is a critical mediator of the senescence program (Alcorta *et al*. 1996). Alternatively, LNCaP cells express AR (Tieszen *et al*. 2011), which also bind mifepristone (Song *et al*. 2004), suggesting that AR may be mediators of mifepristone-induced senescence. Similarly, a senescence-like phenotype was reported in T-47D breast cancer cells exposed to lonaprisan (Busia *et al*. 2011). These data are highly relevant as pro-senescence therapy in cancer is undergoing intense scrutiny (Nardella *et al*. 2011).

### Antiprogestin-induced cell death

When antiprogestins are used at high enough concentrations or for prolonged periods of time, cells that initially arrest in the cell cycle trigger their own death. A large body of evidence indicates that antiprogestin-induced cell death is associated with the following: i) morphological features of apoptosis; ii) downregulation of antiapoptotic Bcl2 family members (e.g. Bcl2 and BclX_L_) and inhibitors of apoptotic proteins (e.g. XIAP); iii) upregulation of pro-apoptotic Bcl2 family members (e.g., Bax); iv) nuclear and DNA fragmentation; and v) downstream activation of caspase 3. For instance, in ovarian cancer, micromolar concentrations of mifepristone, ORG-31710, ulipristal, telapristone, 17α-hydroxy CDB-4124, and CDB-4453 caused cell death with morphological features of apoptosis, accumulation of fragmented hypodiploid DNA, and activation of the executer of apoptosis, caspase 3 (Goyeneche *et al*. 2012, Gamarra-Luques *et al*. 2014). Cleavage of poly(adenosine 5′-diphosphate-ribose) polymerase (PARP), a substrate for active caspase 3, was observed in ovarian cancer cells after exposure to mifepristone, ORG-31710, and ulipristal. Ulipristal, however, at the same time upregulated PARP, a phenomenon that has been previously shown in cultured human uterine leiomyoma cells (Xu *et al*. 2005). In addition, ulipristal upregulated antiapoptotic proteins XIAP and Bcl2, yet cell death ensued with less effectiveness than that caused by mifepristone or ORG-31710, in which XIAP and Bcl2 were downregulated (Goyeneche *et al*. 2012).

In cultured human uterine leiomyoma cells, ulipristal was shown to downregulate the antiapoptotic protein Bcl2 (Xu *et al*. 2005). In the cholangiocarcinoma cell line FRH-0201, mifepristone blocked growth, induced apoptosis, and upregulated pro-apoptotic Bax with a simultaneous downregulation of Bcl2 (Sun *et al*. 2012). In endometrial HEC-1A and Ishikawa cancer cells, Bcl2 levels decreased in response to mifepristone in association with the increase in tumor suppressor p53 (Navo *et al*. 2008). An increase in pro-apoptotic Bax and FAS ligand, and a concomitant decrease in antiapoptotic Bcl2 and activation of caspase 3, was also observed in Ishikawa cells upon treatment with mifepristone (Li *et al*. 2005). Potentiation of apoptotic cell death was observed after exposing Ishikawa endometrial cancer cells to the combined treatment of mifepristone and progesterone (Moe *et al*. 2009). An increase in Bax levels and a decrease in Bcl2 levels were also observed in endometrial Hec-1A, KLE, and RL95-2 when treated with doses of mifepristone that caused apoptosis (Schneider *et al*. 1998). In the endometrial cell line EM42, mifepristone stimulated the activity of the transcription factor nuclear factor kappa B (NFkB) and induced apoptosis mediated by the induction of pro-apoptotic Bax and downregulation of antiapoptotic Bcl2, in a NFkB-dependent manner (Han & Sidell 2003). In HeLa cervical adenocarcinoma cells resistant to mitomycin C, mifepristone increased BAX expression while decreasing expression of BCL2 (Chen *et al*. 2014). In prostate cancer cells, presence of mifepristone sensitized the cells to apoptosis induced by TNFα-related apoptosis inducing ligand (TRAIL) by promoting activation of caspase 8 and truncation of pro-apoptotic Bcl2 family member Bid (Eid *et al*. 2002). Finally, in human SGC-7901 gastric adenocarcinoma cells, mifepristone blocked cell proliferation and induced morphological features of apoptosis in a dose-dependent manner in association with downregulation of pro-survival BclX_L_ and increased caspase 3 activity (Li *et al*. 2004*a*).

### PR and antiprogestin-induced antiproliferation

As several tumors of both gynecological and non-gynecological origin are steroid hormone-dependent and express PR, antiprogestins have been investigated as potential anti-cancer therapeutic agents largely based on their capacity to modulate such receptors. However, the role of PR in the antiproliferative effect of antiprogestins is not without complexity and apparent discrepancies. For instance, mifepristone inhibited the growth of ER-negative/PR-negative MDA-MB-231 breast cancer cells (Liang *et al*. 2003). In another study, mifepristone showed an agonistic effect potentiating progesterone-mediated growth retardation and apoptosis (Moe *et al*. 2009). Such potentiation of cytotoxicity of progesterone by mifepristone was reported also in PR-positive MCF-7 breast cancer cells as well as in PR-negative C4-I cervical carcinoma cells, suggesting that the presence of PR may not be essential for the antigrowth properties of both, progesterone and mifepristone (Fjelldal *et al*. 2010). In the aforementioned studies, however, mifepristone was utilized at micromolar concentrations, suggesting that at such doses the antigrowth effect may utilize a PR-independent mechanism as the concentrations needed to saturate intracellular PR are in the nanomolar range (Nardulli & Katzenellenbogen 1988).

The expression of PR in antiprogestin-responsive cancer cells is also controversial, probably as a consequence of the complex variables involved in PR actions, including the following: i) tissue-specific effects; ii) the presence of two isoforms (PR-A and PR-B) with distinct properties; iii) difficulties in tailoring the mRNA expression levels with that of protein receptor levels due to the scarcity of specific antibodies for each PR isoform; and iv) the differential kinetics of the isoforms, with PR-A being more stable than PR-B, because the latter undergoes post-translational modifications including phosphorylation, ubiquitination, acetylation, and SUMOylation, all contributing to its rapid turnover, and, probably, the difficulty in its detection. These factors contributing to the complexity in PR activity have been recently addressed in a comprehensive review (Hagan & Lange 2014).

In ovarian cancer, the majority of cell lines reported in the literature lack or show very low levels of the canonical PR (Hamilton *et al*. 1984, Keith Bechtel & Bonavida 2001, McDonnel & Murdoch 2001, Akahira *et al*. 2002). Using an antibody that detected PR-A and PR-B isoforms in MCF-7 breast cancer cells, we reported that such receptor isoforms were not found in ovarian (SKOV-3 and OVCAR-3), breast (MDA-MB-231), prostate (LNCaP and PC-3), bone (U-2OS and SAOS-2), and meningioma (IOMM-Lee) cancer cell lines cultured under similar conditions (Tieszen *et al*. 2011). However, these cells lines, regardless of PR expression, responded to the growth inhibitory properties of micromolar doses of mifepristone. Furthermore, the abundance of PR-A and PR-B proteins in MCF-7 was highly reduced upon treatment with mifepristone, which retained its growth inhibition properties, discouraging the role of these nuclear receptors as mediators of the growth inhibitory effect of mifepristone. Similarly, in T-47D breast cancer cells made resistant to aromatase inhibitors, which express high levels of aromatase and grow in response to testosterone, telapristone caused growth arrest in association with downregulation of PR-B mRNA and protein levels (Gupta *et al*. 2013*a*).

The requirement for PR as a mediator of the antigrowth effect of antiprogestins, however, has been shown in other experimental models. For instance, *in vivo* studies on mice with MPA-induced mammary carcinomas, antisense oligodeoxynucleotides against PR that leads to *in vivo* knockdown of the receptor caused inhibition of tumor growth similar to that of mifepristone (Lamb *et al*. 2005). In this model system, PR-A appears as a critical PR isoform conferring sensitivity to antiprogestins, as antiprogestin-resistant variants of the MPA-induced mammary tumors depict a heightened downregulation of PR-A when compared with antiprogestin-sensitive tumors (Wargon *et al*. 2009, Lanari *et al*. 2012). Using primary cultures of cells isolated from the MPA-induced mouse mammary carcinomas, mifepristone blocked MPA-induced growth at nanomolar concentrations (Lamb *et al*. 1999). More recently, it has been demonstrated that in these cells the antiprogestin increased tissue remodeling, which favored the efficacy of nanoparticle carrying chemotherapeutic agents (Sequeira *et al*. 2014). Notably, in this latter work, mifepristone-induced tissue remodeling involved the increase in the vascularity of the tumor and the increase in the ratio of stromal tissue to tumor tissue, indicating the tumor microenvironment as an evident target of antiprogestin therapy. From this mouse model of breast cancer, it has been suggested that tumors with levels of PR-A higher than PR-B should be the ones to be targeted with antiprogestin therapy (Lanari *et al*. 2012).

In human breast cancer cells, PR-B seems to be a critical determinant of the responsiveness to the antiproliferative effect of antiprogestins (reviewed in Knutson & Lange (2014)). The development of T-47D cells overexpressing either PR-A or PR-B led to the conclusion that, in the presence of mifepristone, there is inappropriate transactivation of PR-B but not of PR-A (Sartorius *et al*. 1994). In the presence of a ligand, PR-B is phosphorylated at Ser294 and translocated to the nucleus where it operates as a highly active transcription factor triggering gene expression encoding for proteins needed for cell cycle progression, proliferation, and survival (cyclin D1, Myc and Bcl2 respectively). However, when activated, PR-B has a very short half-life as phospho-Ser294-PR-B is recognized for degradation by the ubiquitin–proteasome system making activated PR-B difficult to detect by western blotting (Knutson & Lange 2014). Thus, in the studies described earlier using mifepristone as a growth inhibitor in human cancer cell lines, the fact that PR is undetectable by western blot (Tieszen *et al*. 2011) does not rule out its presence as, under the culture conditions used, it could have had a heightened turnover that did not allow for its detection with the antibodies utilized.

Early in 1987, experimental evidence using breast cancer cells suggested that the antihormone and antiproliferative activity of antiprogestin mifepristone are dissociated (Bardon *et al*. 1987). Our previous analysis of the literature has suggested that such statement is still valid and further studies need to be conducted to find out the role of PR in antiprogestin-mediated antiproliferative activity.

### Membrane PR

The antitumor effect of antiprogestins may well be mediated by non-cognate PR, such as membrane PRs (mPRα, β, γ, δ, and ε; Thomas *et al*. 2007, Gellersen *et al*. 2009, Dressing *et al*. 2011). This hypothesis is supported by studies on *Xenopus* oocytes in which progesterone promotes germinal vesicle breakdown (GVBD) – an indicator of meiotic maturation – probably due to the interplay of cognate intracellular PR and mPR (Josefsberg Ben-Yehoshua *et al*. 2007). In this model system, antiprogestin mifepristone depicted a progesterone-like effect at micromolar concentrations (Sadler *et al*. 1985). When these mPRs were expressed in yeast, antiprogestin mifepristone also had an agonist effect (Smith *et al*. 2008). Curiously, *Xenopus* intracellular PR lacks the glycine residue considered essential for intracellular PR binding to mifepristone (Benhamou *et al*. 1992), suggesting that the membrane-linked but not the classical intracellular PR mediates the progesterone-like mifepristone-induced GVBD. Thus, effects of antiprogestins mediated via mPR might need to be differentiated from those controlled via the classical PR.

Membrane PRs have been shown in human breast biopsies, in cognate PR-positive MCF-7 and SKBR3 breast cancer cell lines, in cognate PR-negative MDA-MB-468 breast cancer cells, in HeLa cervical cancer cells, and in ovarian cancer biopsies (reviewed in Dressing *et al*. (2011)). Ovarian cancer cell lines (SKOV-3 and ES2) express the mRNA of the three mPRs, which regulate PKA, p38, and JNK signaling pathways (Dressing *et al*. 2011).

Another non-cognate PR, termed PR membrane component 1 (PGRMC1), was shown to increase in advanced ovarian cancer in association with an absence of the classical PR. Furthermore, it was shown that overexpression of PGRMC1 interfered with cisplatin-induced cytotoxicity, which suggests that PGRMC1 has a survival role in this particular gynecological cancer (Peluso *et al*. 2008).

In summary, the roles of mPR and PGRMC1 in antiprogestin-mediated antiproliferation in cells involved in gynecological diseases represent an attractive area of research that could lead to novel therapeutic interventions.

### Growth factors and signaling pathways involved in antiprogestin-mediated growth inhibition

Transforming growth factor beta 1 (TGFβ1) is induced by mifepristone and triggers apoptosis in LNCaP-C4 prostate cancer cells (Liang *et al*. 2002). In ER-negative/PR-negative MDA-MB-231 breast cancer cells, mifepristone and 4-hydroxytamoxifen potentiated one another's effect in inducing apoptosis associated with DNA fragmentation and cytochrome *c* release from the mitochondrial compartment and activation of downstream executer of apoptosis, caspase 3, mediated via upregulation of TGFβ1 (Liang *et al*. 2003). Furthermore, MCF-7 and T-47D PR-positive breast cancer cell lines produce more TGFβ when incubated in the presence of onapristone in association with growth inhibition (Dannecker *et al*. 1996). Conversely, for insulin-like growth factor1 IGF1), it was shown that it attenuates antiprogestin-mediated apoptosis in ER-positive breast cancer cells (Periyasamy-Thandavan *et al*. 2012). Ulipristal was shown to block the expression of fibronectin and *VEGFA* mRNA induced by activin A in cultured leiomyoma cells, thus blocking cell growth (Ciarmela *et al*. 2014) and the substantial angiogenesis required by these tumors to proliferate (Xu *et al*. 2006). In MPA-induced mouse mammary tumor line C4-HD, mifepristone was able to block the proliferation induced by bFGF alone or the combination bFGF/MPA (Lamb *et al*. 1999). Mifepristone blocked the secretion of IGF1 induced by progesterone and E_2_ in *ex vivo* explants of ER-positive/PR-positive breast cancers (Milewicz *et al*. 2005).

In MDA-MB-231 breast cancer cells transfected with PR, mifepristone arrested the cells in the G0/G1 phase of the cell cycle while activating p44/p42 MAPK (Lin *et al*. 2001). In another study, MAPK inhibitors and antiprogestins blocked the growth of BT-474 breast cancer cells upon induction with EGF and progestins (Knutson *et al.* 2012). In cultured mouse cancer cells isolated from mammary tumors induced by MPA, mifepristone, onapristone, and lonaprisan blocked proliferation induced by MPA or FGF2 while increasing phosphorylation of ERK via rapid mechanisms (reviewed in Lanari *et al*. (2012)). When cells from previous tumors were maintained in 3D cultures, lonaprisan induced cell death more efficiently in MPA-dependent cells having a low AKT activity, suggesting the survival role of the PI3K/Akt pathway in these cancer cells (Polo *et al*. 2010). In ovarian cancer cells cultured either in 2D or 3D, cytostatic doses of mifepristone caused synergistic lethality when combined with an inhibitor of the PI3K/Akt survival pathway, in association with downregulation of antiapoptotic proteins BCL2 and XIAP, and cleavage of PARP (Wempe *et al*. 2013).

Another pathway involved in antiprogestin-mediated growth inhibition is the Wnt pathway, which is critically involved in cancer development (reviewed in Veeck & Dahl (2012) and Gupta *et al*. (2013*b*)). For instance, Wnt1 was blocked by mifepristone in MCF-7 cells, while overexpression of Wnt1 prevented mifepristone-induced growth inhibition (Benad *et al*. 2011).

Mounting evidence demonstrates the role of cytoplasmic pro-proliferative protein kinases such as MARK, CK2, and Cdk2 in controlling the phosphorylation status of classic PR (reviewed in Trevino & Weigel (2013)). Cdk2 is a cell cycle kinase critically important for the hyperphosphorylation of Rb, thus allowing the detachment of E2F transcription factor from Rb, and making E2F available to regulate the expression of genes driving DNA synthesis during the S phase (Conradie *et al*. 2010). Thus, small molecules are under development for the blockage of Cdks, among them Cdk2, to treat cancer (reviewed in Esposito *et al*. (2013)). In human cells spanning many cancer types, we have shown that alongside blocking proliferation, mifepristone strongly inhibited the activity of Cdk2 (Tieszen *et al*. 2011). In ovarian cancer cells, we also reported that mifepristone promoted the upregulation of p21^cip1^ and p27^kip1^ and their association with Cdk2 in the nuclear compartment, thus blunting the activity of Cdk2 otherwise required to drive G1/S cell cycle progression (Goyeneche *et al*. 2007, 2012). Cdk2 is critically important to phosphorylate PR at Ser400, thus activating the transcriptional activity of PR in a ligand-independent manner during the cell cycle. As Cdk2 activity is blocked by p27^kip1^ overexpression (Pierson-Mullany & Lange 2004), we propose that mifepristone-induced cell cycle arrest in G1 is mediated by p27^kip1^-induced Cdk2 inhibition upstream of PR activation, thus preventing ligand-independent PR transcriptional activity required for cell cycle progression.

### Glucocorticoid receptors

Except for the new generation of antiprogestins that bind GR with a much less affinity than PR, older antiprogestins such as mifepristone bind GR with a high affinity (Mao *et al*. 1992). Furthermore, GR are ubiquitously expressed in normal as well as cancer cells (Agarwal 1996)). Our laboratory reported abundant expression of GR isoforms alpha (GRα) and beta (GRβ) as measured by western blot in ovarian, breast, prostate, bone, and brain cancer cells (Tieszen *et al*. 2011, Telleria & Goyeneche 2012). All such cell lines studied responded to mifepristone with growth inhibition, whereas the relative expression of GRα and GRβ was very variable, yet did not show any significant correlation with the growth inhibition potency of mifepristone (Tieszen *et al*. 2011). In OV2008 cancer cells, mifepristone, ORG-31710, and ulipristal increased p21^cip1^ and p27^kip1^ and caused cell cycle arrest without major changes in the expression of the GR isoforms. Yet, under the same experimental conditions, equimolar concentrations of the GR agonist dexamethasone did not cause growth arrest or upregulation of p21^cip1^ and p27^kip1^, but blunted the expression of GR (Telleria & Goyeneche 2012). By contrast, in three endometrial cancer cell lines (Hec-1A, LEK, and RL95-2), mifepristone inhibited cell proliferation while causing a decline in the abundance of GR (Schneider *et al*. 1998). Mostly, on GRα, mifepristone has an antagonistic activity; yet, it was shown to have an agonistic potency depending on the concentration of GR in the cell (Zhang *et al*. 2007). On the other hand, although GRβ has been considered a dominant negative regulator of GRα (Oakley *et al*. 1999, Yudt *et al*. 2003, Taniguchi *et al*. 2010), it was also reported to function, in the absence of GRα, as a receptor for mifepristone, leading to nuclear translocation and transcriptional activity (Lewis-Tuffin *et al*. 2007). All cancer cell lines we exposed to mifepristone undergo proliferation inhibition with IC50s – concentrations that reduce growth by 50% – ranging from 9 to 30 μm, and all cells had high expression of GRβ, yet variable abundances of GRα fluctuating from no expression (LNCaP) to high expression (SKOV-3 and MDA-MB-231) (Tieszen *et al*. 2011, Telleria & Goyeneche 2012). These evidences suggest to us that the role of mainly GRβ on antiprogestin-mediated cell growth inhibition deserves further exploration.

### Antioxidation

Early in 1994, it was reported that mifepristone, when used at micromolar doses, operates as an antioxidant and that such activity resided in the dimethylaminophenyl side chain of the molecule (Parthasarathy *et al*. 1994), which is present in most of the antiprogestins developed to date. In support of this action connected with the antiproliferative effect of mifepristone, two reports attributed such a mechanism in endometrial cells and macrophages (Roberts *et al*. 1995, Murphy *et al*. 2000). More recently, in a study in which the administration of 50 mg mifepristone every other day for 12 weeks before surgery led to leiomyoma volume reduction, when tissues were studied after surgery, it was clear that the glutathione pathway was the most clearly altered pathway. In particular, the antioxidant enzyme glutathione-S-transferase mu1 (GSTM1), reported to offer protection against free radicals and products of oxidation stress (Sharma *et al*. 2004), was significantly overexpressed among the good responders compared with the non-responders (Engman *et al*. 2013). The authors suggest that this enzyme might be important in the regulation of pathways leading to inhibition of cell cycle progression or to facilitate apoptosis. GSTM1 can be a potential molecular marker of objective response to mifepristone therapy. Furthermore, G1 arrest and p21^cip^ upregulation were shown to be amplified in response to antioxidants in a p53-independent manner (Liu *et al*. 1999, Liberto & Cobrinik 2000). This mechanism can explain our results in which mifepristone blocked the growth of ovarian cancer cells regardless of their p53 expression background (Goyeneche *et al*. 2007, Freeburg *et al*. 2009*b*).

### Endoplasmic reticulum stress

Cancer cells, when compared with non-cancer cells, operate with increased expression of endoplasmic reticulum stress-related proteins, a phenomenon termed ‘endoplasmic reticulum aggravation’ as a consequence of the environment within which cancer cells usually proliferate: reduced nutrients, acidosis, energy deficiency, and hypoxia (reviewed in Schonthal (2013)). First, in 2007, it was shown that asoprisnil triggered endoplasmic reticulum stress-induced apoptosis in cultured human uterine leiomyoma cells (Xu *et al*. 2007). Secondly, a serendipitous study carried out in 2010 reported that mifepristone induced endoplasmic reticulum stress in non-small cell lung carcinoma cells (Dioufa *et al*. 2010). Using genomic and proteomic screenings, we have recently reported that cytostatic concentrations of antiprogestin mifepristone trigger the unfolded protein response (UPR; Hapon *et al*. 2013). The UPR is a mechanism geared to compensate for the stress and to promote cell survival, but, if overwhelmed, it triggers a cell death pathway (Hetz 2012, Urra *et al*. 2013). We showed that the master chaperone involved in the UPR and associated with cell survival, glucose-regulated protein (GRP) of 78 KDa (GRP78) increased in response to mifepristone in a dose- and time-dependent manner, and independently of p53 tumor suppressor and sensitivity to chemotherapeutic agent cisplatin. In addition, we found the transcription factor C/EBP homologous protein (CHOP) to be highly upregulated, the induction of which is usually linked to cell death. We hypothesize that the UPR integrates the cytotoxicity of antiprogestins toward cancer cells when used as monotherapy or in combination therapies triggering, respectively, cell cycle arrest (cytostasis) or cell death (lethality) depending on the degree of cellular stress generated.

### Cytoskeleton, adhesion, migration, and invasion

One key component of cancer metastasis is the detachment or de-adhesion of cancer cells from one tissue, migration, and invasion through the extracellular matrix, and re-adhesion to a nearby or distant location. Considering that progestins regulate metastasis-related molecules, it was proposed that antiprogestins could be relevant to fight metastatic diseases (Shi *et al*. 1994). In breast cancer cells expressing PR-B, it was shown that mifepristone blocked the migration induced by IGF1 (Ibrahim *et al*. 2008). We have recently reported using SKOV-3 ovarian cancer cells, MDA-MB-231 breast cancer cells, U87MG glioblastoma cells, and LNCaP prostate cancer cells that a concentration of antiprogestin mifepristone sufficient to block cell proliferation caused changes in the cellular structure with cells developing a thin cytoplasm with neurite-like protrusions. Such changes were associated with redistribution of cytoskeletal actin fibers that mainly form non-adhesive membrane ruffles, which are sheet-like membrane folds that do not attach to the extracellular matrix, leading to a decline in the capacity of the cell to adhere to extracellular substrates (Brandhagen *et al*. 2013). This morphological phenomenon was associated with diminished cellular migration and invasion capacities toward extracellular matrix (A A Goyeneche, B N Brandhagen, R Srinivasan, and C M Telleria, unpublished observations). In the human gastric MKN-45 adenocarcinoma cells, mifepristone, in a dose-dependent manner, inhibited their adhesion to extracellular matrix and reduced migration through 8 μm pore size membrane filters; *in vivo*, 8 week treatment with mifepristone reduced the number of distant lung foci in nude mice carrying subcutaneous tumor xenografts (Li *et al*. 2004*b*). Consistent with these data, in Ishikawa endometrial cancer cells receiving mifepristone for 12 h, it was shown by RNA sequencing that mifepristone downregulated genes associated with cell–cell contact and adhesion (Tamm-Rosenstein *et al*. 2013). More recently, a monodemethylated metabolite of mifepristone termed metapristone (RU-42633) has been shown to block the adhesion of human colon cancer HT-29 cells to endothelial cells (Wang *et al*. 2014). These initial data provide the basis for further studies on the antimetastatic properties of antiprogestins.

### Antiprogestins in clinical trials for gynecological diseases

Table 1 depicts the ongoing clinical trials registered in the public access database maintained by the US National Library of Medicine at the National Institutes of Health (NIH) (http://ClinicalTrials.gov). Of notice is the number of ongoing studies using mifepristone for conditions such as leiomyoma, breast, ovarian, and endometrial cancers; ulipristal for leiomyoma; and telapristone for endometriosis. The results of these trials will be essential in moving forward the utilization of antiprogestins as adjuvant treatment for gynecological diseases should they confirm their potentiality reflected in the preclinical and clinical studies detailed above. The progress made in the past years exploring the treatment of gynecological conditions such as endometriosis and leiomyoma has been remarkable; yet, the consequence has been of a less emphasis on treating gynecological cancers. However, as new information evolves on the pathogenesis of gynecological cancers and the mechanisms of action of available antiprogestins, there is optimism for quick developments in bringing these compounds to the clinic. Antiprogestins can ameliorate the signs and symptoms, and prevent (as monotherapy) or trigger chronic remission (as adjuvant to standard chemotherapeutic agents) of gynecological malignancies.

## Concluding remarks

Since the synthesis of mifepristone in 1981, much progress has been made in understanding the mechanisms whereby antiprogestins act at the tissue, cellular, and molecular levels in both normal cells and in cells with a derangement of their proliferation capacity. There has been a concurrent development of new compounds, major progress in understanding the biology of PR and GR isoforms, their cellular localization within the context of the molecular environment, and their involvement in driving cell cycle progression and cell death. As the basic molecular biology of steroidal compounds evolves, the applications of compounds designed to bind PR should be recalibrated continuously. For instance, as reviewed herein, we should exploit their potential for treating gynecological conditions related to unbalanced cell proliferation. The timeframe for their usage on such conditions, as well as their effective dosage, should be addressed to prevent undesired side effects. The analysis of the molecular genetics of the disease will be critical for identifying the cohort of individuals who more probably will benefit from antiprogestin treatment and, by doing so, prevent the inclusion of patients not likely to respond. Inappropriate inclusion of patients can highly contribute to derailing the success of the clinical studies. With the current ongoing clinical trials, special attention should be given to patients that present an objective response to the treatment despite the fact that they may not represent a significant group within the trial. Such responders should guide the identification of the optimal conditions for objective responses of disease cells with respect to normal cells, such as the ratio of expression of PR-A/PR-B, levels of GRα and GRβ, intracellular signal transduction environment, oxidative stress, and excess or lack of critical cell cycle regulatory proteins to mention some. Thus, for instance, despite that the clinical trials for the usage of antiprogestin mifepristone against ovarian and breast cancers have not been as encouraging as originally envisioned, the knowledge gained on the molecular underpinning of such patients should lead to a better patient selection while more closely bridging basic and translational research.

## Supplementary Material

http://www.reproduction-online.org/content/149/1/R15/suppl/DC1

## Figures and Tables

**Table 1:** Current active interventional clinical trials using antiprogestins for gynecological diseases registered in ClinicalTrials.gov^a^.

**Antiprogestin**	**Gynecological disease**	**Phase**	**Clinical trial ID**
Mifepristone (RU-38486)	Leiomyoma	III	NCT00133705
		I	NCT00579475
		II	NCT00881140
II/III	NCT00712595
	Breast cancer	I	NCT01493310
		II	NCT01898312
	Breast and ovarian cancers	I	NCT02046421
I	NCT02014337
	Endometrial cancer	II	NCT00505739
Ulipristal (CDB-2914)	Leiomyoma	II	NCT00044876
III	NCT02147158
		III	NCT01642472
		III	NCT01629563
Telapristone (CDB-4124)	Endometriosis	II	NCT01728454
Breast cancer	II	NCT01800422
Onapristone (ZK-98299)	PR expressing cancers	I	NCT02052158

a Data obtained August 5, 2014.

## References

[bib1] Achille C, Sundaresh S, Chu B, Hadjiargyrou M (2012). Cdk2 silencing via a DNA/PCL electrospun scaffold suppresses proliferation and increases death of breast cancer cells. PLoS ONE.

[bib2] Afhuppe W, Beekman JM, Otto C, Korr D, Hoffmann J, Fuhrmann U, Moller C (2010). *In vitro* characterization of ZK 230211 – a type III progesterone receptor antagonist with enhanced antiproliferative properties. Journal of Steroid Biochemistry and Molecular Biology.

[bib3] Agarwal MK (1996). The antiglucocorticoid action of mifepristone. Pharmacology & Therapeutics.

[bib4] Akahira J, Suzuki T, Ito K, Kaneko C, Darnel AD, Moriya T, Okamura K, Yaegashi N, Sasano H (2002). Differential expression of progesterone receptor isoforms A and B in the normal ovary, and in benign, borderline, and malignant ovarian tumours. Japanese Journal of Cancer Research.

[bib5] Alcorta DA, Xiong Y, Phelps D, Hannon G, Beach D, Barrett JC (1996). Involvement of the cyclin-dependent kinase inhibitor p16 (INK4a) in replicative senescence of normal human fibroblasts. PNAS.

[bib6] Bakker GH, Setyono-Han B, Henkelman MS, de Jong FH, Lamberts SW, van der Schoot P, Klijn JG (1987). Comparison of the actions of the antiprogestin mifepristone (RU486), the progestin megestrol acetate, the LHRH analog buserelin, and ovariectomy in treatment of rat mammary tumours. Cancer Treatment Reports.

[bib7] Bakker GH, Setyono-Han B, Portengen H, De Jong FH, Foekens JA, Klijn JG (1989). Endocrine and antitumor effects of combined treatment with an antiprogestin and antiestrogen or luteinizing hormone-releasing hormone agonist in female rats bearing mammary tumours. Endocrinology.

[bib8] Bardon S, Vignon F, Chalbos D, Rochefort H (1985). RU486, a progestin and glucocorticoid antagonist, inhibits the growth of breast cancer cells via the progesterone receptor. Journal of Clinical Endocrinology and Metabolism.

[bib9] Bardon S, Vignon F, Montcourrier P, Rochefort H (1987). Steroid receptor-mediated cytotoxicity of an antiestrogen and an antiprogestin in breast cancer cells. Cancer Research.

[bib10] Baulieu EE (1991). On the mechanism of action of RU486. Annals of the New York Academy of Sciences.

[bib11] Baulieu EE (1997). RU 486 (mifepristone). A short overview of its mechanisms of action and clinical uses at the end of 1996. Annals of the New York Academy of Sciences.

[bib12] Belanoff JK, Blasey CM, Clark RD, Roe RL (2010). Selective glucocorticoid receptor (type II) antagonist prevents and reverses olanzapine-induced weight gain. Diabetes, Obesity & Metabolism.

[bib13] Benad P, Rauner M, Rachner TD, Hofbauer LC (2011). The anti-progestin RU-486 inhibits viability of MCF-7 breast cancer cells by suppressing WNT1. Cancer Letters.

[bib14] Benagiano G, Bastianelli C, Farris M (2008). Selective progesterone receptor modulators 3: use in oncology, endocrinology and psychiatry. Expert Opinion on Pharmacotherapy.

[bib15] Benhamou B, Garcia T, Lerouge T, Vergezac A, Gofflo D, Bigogne C, Chambon P, Gronemeyer H (1992). A single amino acid that determines the sensitivity of progesterone receptors to RU486. Science.

[bib16] Bernardi LA, Pavone ME (2013). Endometriosis: an update on management. Women's Health.

[bib17] Bowden RT, Hissom JR, Moore MR (1989). Growth stimulation of T47D human breast cancer cells by the anti-progestin RU486. Endocrinology.

[bib18] Brandhagen BN, Tieszen CR, Ulmer TM, Tracy MS, Goyeneche AA, Telleria CM (2013). Cytostasis and morphological changes induced by mifepristone in human metastatic cancer cells involve cytoskeletal filamentous actin reorganization and impairment of cell adhesion dynamics. BMC Cancer.

[bib19] Brown KA, Roberts RL, Arteaga CL, Law BK (2004). Transforming growth factor-β induces Cdk2 relocalization to the cytoplasm coincident with dephosphorylation of retinoblastoma tumor suppressor protein. Breast Cancer Research.

[bib20] Busia L, Faus H, Hoffmann J, Haendler B (2011). The antiprogestin Lonaprisan inhibits breast cancer cell proliferation by inducing p21 expression. Molecular and Cellular Endocrinology.

[bib21] Bygdeman M, Swahn ML (1985). Progesterone receptor blockage. Effect on uterine contractility and early pregnancy. Contraception.

[bib22] Cadepond F, Ulmann A, Baulieu EE (1997). RU486 (mifepristone): mechanisms of action and clinical uses. Annual Review of Medicine.

[bib23] Cancer Genome Atlas Network (2012). Comprehensive molecular portraits of human breast tumours. Nature.

[bib24] Cancer Genome Atlas Research Network (2011). Integrated genomic analyses of ovarian carcinoma. Nature.

[bib25] Chabbert-Buffet N, Meduri G, Bouchard P, Spitz IM (2005). Selective progesterone receptor modulators and progesterone antagonists: mechanisms of action and clinical applications. Human Reproduction Update.

[bib26] Chen W, Ohara N, Wang J, Xu Q, Liu J, Morikawa A, Sasaki H, Yoshida S, Demanno DA, Chwalisz K (2006). A novel selective progesterone receptor modulator asoprisnil (J867) inhibits proliferation and induces apoptosis in cultured human uterine leiomyoma cells in the absence of comparable effects on myometrial cells. Journal of Clinical Endocrinology and Metabolism.

[bib27] Chen H, Duan J, Zuo F (2014). Mechanism of the reversal effect of mifepristone on drug resistance of the human cervical cancer cell line HeLa/MMC. Genetics and Molecular Research.

[bib28] Chwalisz K, Larsen L, Mattia-Goldberg C, Edmonds A, Elger W, Winkel CA (2007). A randomized, controlled trial of asoprisnil, a novel selective progesterone receptor modulator, in women with uterine leiomyomata. Fertility and Sterility.

[bib29] Ciarmela P, Carrarelli P, Islam MS, Janjusevic M, Zupi E, Tosti C, Castellucci M, Petraglia F (2014). Ulipristal acetate modulates the expression and functions of activin A in leiomyoma cells. Reproductive Sciences.

[bib30] Clark RD (2008). Glucocorticoid receptor antagonists. Current Topics in Medicinal Chemistry.

[bib31] Classen S, Possinger K, Pelka-Fleischer R, Wilmanns W (1993). Effect of onapristone and medroxyprogesterone acetate on the proliferation and hormone receptor concentration of human breast cancer cells. Journal of Steroid Biochemistry and Molecular Biology.

[bib32] Coleman RL, Monk BJ, Sood AK, Herzog TJ (2013). Latest research and treatment of advanced-stage epithelial ovarian cancer. Nature Reviews. Clinical Oncology.

[bib33] Colucci JK, Ortlund EA (2013). X-ray crystal structure of the ancestral 3-ketosteroid receptor–progesterone–mifepristone complex shows mifepristone bound at the coactivator binding interface. PLoS ONE.

[bib34] Conradie R, Bruggeman FJ, Ciliberto A, Csikasz-Nagy A, Novak B, Westerhoff HV, Snoep JL (2010). Restriction point control of the mammalian cell cycle via the cyclin E/Cdk2:p27 complex. FEBS Journal.

[bib35] Dannecker C, Possinger K, Classen S (1996). Induction of TGF-β by an antiprogestin in the human breast cancer cell line T-47D. Annals of Oncology.

[bib36] De Bosscher K (2010). Selective glucocorticoid receptor modulators. Journal of Steroid Biochemistry and Molecular Biology.

[bib37] Dioufa N, Kassi E, Papavassiliou AG, Kiaris H (2010). Atypical induction of the unfolded protein response by mifepristone. Endocrine.

[bib38] Donnez J, Tomaszewski J, Vazquez F, Bouchard P, Lemieszczuk B, Baro F, Nouri K, Selvaggi L, Sodowski K, Bestel E (2012). Ulipristal acetate versus leuprolide acetate for uterine fibroids. New England Journal of Medicine.

[bib39] Dressing GE, Goldberg JE, Charles NJ, Schwertfeger KL, Lange CA (2011). Membrane progesterone receptor expression in mammalian tissues: a review of regulation and physiological implications. Steroids.

[bib40] Eid MA, Lewis RW, Kumar MV (2002). Mifepristone pretreatment overcomes resistance of prostate cancer cells to tumor necrosis factor α-related apoptosis-inducing ligand (TRAIL). Molecular Cancer Therapeutics.

[bib41] Eisinger SH, Meldrum S, Fiscella K, le Roux HD, Guzick DS (2003). Low-dose mifepristone for uterine leiomyomata. Obstetrics and Gynecology.

[bib42] Eisinger SH, Fiscella J, Bonfiglio T, Meldrum S, Fiscella K (2009). Open-label study of ultra low-dose mifepristone for the treatment of uterine leiomyomata. European Journal of Obstetrics, Gynecology, and Reproductive Biology.

[bib43] El Etreby MF, Liang Y, Wrenn RW, Schoenlein PV (1998). Additive effect of mifepristone and tamoxifen on apoptotic pathways in MCF-7 human breast cancer cells. Breast Cancer Research and Treatment.

[bib44] El Etreby MF, Liang Y, Johnson MH, Lewis RW (2000). Antitumor activity of mifepristone in the human LNCaP, LNCaP-C4, and LNCaP-C4-2 prostate cancer models in nude mice. Prostate.

[bib45] Ellertson C, Waldman SN (2001). The mifepristone–misoprostol regimen for early medical abortion. Current Women's Health Reports.

[bib46] Engman M, Skoog L, Soderqvist G, Gemzell-Danielsson K (2008). The effect of mifepristone on breast cell proliferation in premenopausal women evaluated through fine needle aspiration cytology. Human Reproduction.

[bib47] Engman M, Granberg S, Williams AR, Meng CX, Lalitkumar PG, Gemzell-Danielsson K (2009). Mifepristone for treatment of uterine leiomyoma. A prospective randomized placebo controlled trial. Human Reproduction.

[bib48] Engman M, Varghese S, Lagerstedt Robinson K, Malmgren H, Hammarsjo A, Bystrom B, Lalitkumar PG, Gemzell-Danielsson K (2013). GSTM1 gene expression correlates to leiomyoma volume regression in response to mifepristone treatment. PLoS ONE.

[bib49] Esposito L, Indovina P, Magnotti F, Conti D, Giordano A (2013). Anticancer therapeutic strategies based on CDK inhibitors. Current Pharmaceutical Design.

[bib50] Etemadmoghadam D, Au-Yeung G, Wall M, Mitchell C, Kansara M, Loehrer E, Batzios C, George J, Ftouni S, Weir BA (2013). Resistance to CDK2 inhibitors is associated with selection of polyploid cells in CCNE1-amplified ovarian cancer. Clinical Cancer Research.

[bib51] Fauvet R, Dufournet Etienne C, Poncelet C, Bringuier AF, Feldmann G, Darai E (2006). Effects of progesterone and anti-progestin (mifepristone) treatment on proliferation and apoptosis of the human ovarian cancer cell line, OVCAR-3. Oncology Reports.

[bib52] Fjelldal R, Moe BT, Orbo A, Sager G (2010). MCF-7 cell apoptosis and cell cycle arrest: non-genomic effects of progesterone and mifepristone (RU-486). Anticancer Research.

[bib53] Fleseriu M, Biller BM, Findling JW, Molitch ME, Schteingart DE, Gross C, SEISMIC Study Investigators (2012). Mifepristone, a glucocorticoid receptor antagonist, produces clinical and metabolic benefits in patients with Cushing's syndrome. Journal of Clinical Endocrinology and Metabolism.

[bib54] Freeburg EM, Goyeneche AA, Telleria CM (2009a). Mifepristone abrogates repopulation of ovarian cancer cells in between courses of cisplatin treatment. International Journal of Oncology.

[bib55] Freeburg EM, Goyeneche AA, Seidel EE, Telleria CM (2009b). Resistance to cisplatin does not affect sensitivity of human ovarian cancer cell lines to mifepristone cytotoxicity. Cancer Cell International.

[bib56] Gaddy VT, Barrett JT, Delk JN, Kallab AM, Porter AG, Schoenlein PV (2004). Mifepristone induces growth arrest, caspase activation, and apoptosis of estrogen receptor-expressing, antiestrogen-resistant breast cancer cells. Clinical Cancer Research.

[bib57] Gamarra-Luques CD, Goyeneche AA, Hapon MB, Telleria CM (2012). Mifepristone prevents repopulation of ovarian cancer cells escaping cisplatin-paclitaxel therapy. BMC Cancer.

[bib58] Gamarra-Luques CD, Hapon MB, Goyeneche AA, Telleria CM (2014). Resistance to cisplatin and paclitaxel does not affect the sensitivity of human ovarian cancer cells to antiprogestin-induced cytotoxicity. Journal of Ovarian Research.

[bib59] Gellersen B, Fernandes MS, Brosens JJ (2009). Non-genomic progesterone actions in female reproduction. Human Reproduction Update.

[bib60] Gil JF, Augustine TN, Hosie MJ (2013). Anastrozole and RU486: effects on estrogen receptor α and Mucin 1 expression and correlation in the MCF-7 breast cancer cell line. Acta Histochemica.

[bib61] Gill PG, Vignon F, Bardon S, Derocq D, Rochefort H (1987). Difference between R5020 and the antiprogestin RU486 in antiproliferative effects on human breast cancer cells. Breast Cancer Research and Treatment.

[bib62] Giudice LC, Kao LC (2004). Endometriosis. Lancet.

[bib63] Giulianelli S, Molinolo A, Lanari C (2013). Targeting progesterone receptors in breast cancer. Vitamins and Hormones.

[bib64] Goyeneche AA, Caron RW, Telleria CM (2007). Mifepristone inhibits ovarian cancer cell growth *in vitro* and *in vivo*. Clinical Cancer Research.

[bib65] Goyeneche AA, Seidel EE, Telleria CM (2012). Growth inhibition induced by antiprogestins RU-38486, ORG-31710, and CDB-2914 in ovarian cancer cells involves inhibition of cyclin dependent kinase 2. Investigational New Drugs.

[bib66] Gross C, Blasey CM, Roe RL, Belanoff JK (2010). Mifepristone reduces weight gain and improves metabolic abnormalities associated with risperidone treatment in normal men. Obesity.

[bib67] SGO Clinical Practice Endometrial Cancer Working Group, Burke WM, Orr J, Leitao M, Salom E, Gehrig P, Olawaiye AB, Brewer M, Boruta D, Herzog TJ (2014a). Endometrial cancer: a review and current management strategies: Part II. Gynecologic Oncology.

[bib68] SGO Clinical Practice Endometrial Cancer Working Group, Burke WM, Orr J, Leitao M, Salom E, Gehrig P, Olawaiye AB, Brewer M, Boruta D, Villella J (2014b). Endometrial cancer: a review and current management strategies: Part I. Gynecologic Oncology.

[bib69] Grow DR, Williams RF, Hsiu JG, Hodgen GD (1996). Antiprogestin and/or gonadotropin-releasing hormone agonist for endometriosis treatment and bone maintenance: a 1-year primate study. Journal of Clinical Endocrinology and Metabolism.

[bib70] Grunberg SM, Weiss MH, Russell CA, Spitz IM, Ahmadi J, Sadun A, Sitruk-Ware R (2006). Long-term administration of mifepristone (RU486): clinical tolerance during extended treatment of meningioma. Cancer Investigation.

[bib71] Gupta A, Mehta R, Alimirah F, Peng X, Murillo G, Wiehle R, Mehta RG (2013a). Efficacy and mechanism of action of Proellex, an antiprogestin in aromatase overexpressing and Letrozole resistant T47D breast cancer cells. Journal of Steroid Biochemistry and Molecular Biology.

[bib72] Gupta A, Verma A, Mishra AK, Wadhwa G, Sharma SK, Jain CK (2013b). The Wnt pathway: emerging anticancer strategies. Recent Patents on Endocrine, Metabolic & Immune Drug Discovery.

[bib73] Hagan CR, Lange CA (2014). Molecular determinants of context-dependent progesterone receptor action in breast cancer. BMC Medicine.

[bib74] Hagan CR, Daniel AR, Dressing GE, Lange CA (2012). Role of phosphorylation in progesterone receptor signaling and specificity. Molecular and Cellular Endocrinology.

[bib75] Haie-Meder C, Morice P, Castiglione M, EGW Group (2010). Cervical cancer: ESMO Clinical Practice Guidelines for diagnosis, treatment and follow-up. Annals of Oncology.

[bib76] Hamilton TC, Behrens BC, Louie KG, Ozols RF (1984). Induction of progesterone receptor with 17 β-estradiol in human ovarian cancer. Journal of Clinical Endocrinology and Metabolism.

[bib77] Han S, Sidell N (2003). RU486-induced growth inhibition of human endometrial cells involves the nuclear factor-kappa B signaling pathway. Journal of Clinical Endocrinology and Metabolism.

[bib78] Hansen KA, Eyster KM (2006). A review of current management of endometriosis in 2006: an evidence-based approach. South Dakota Medicine.

[bib79] **Hapon MB, Gamarra-Luques CD, Goyeneche AA, Callegari EA, Eyster KM, Telleria CM** 2013 Induction of the unfolded protein response in ovarian cancer cells exposed to cytostatic concentrations of antiprogestin/antiglucocorticoid mifepristone. *Prooceedings of the American Association for Cancer Research, Special Conference on Advances in Ovarian Cancer Research: From Concept to Clinic*. **A23**. Miami, USA.

[bib80] Helguero LA, Viegas M, Asaithamby A, Shyamala G, Lanari C, Molinolo AA (2003). Progesterone receptor expression in medroxyprogesterone acetate-induced murine mammary carcinomas and response to endocrine treatment. Breast Cancer Research and Treatment.

[bib81] Herrmann W, Wyss R, Riondel A, Philibert D, Teutsch G, Sakiz E, Baulieu EE (1982). The effects of an antiprogesterone steroid in women: interruption of the menstrual cycle and of early pregnancy. Comptes rendus des séances de l'Académie des sciences. Série III, Sciences de la vie.

[bib82] Hetz C (2012). The unfolded protein response: controlling cell fate decisions under ER stress and beyond. Nature Reviews. Molecular Cell Biology.

[bib83] Horwitz KB (1985). The antiprogestin RU38 486: receptor-mediated progestin versus antiprogestin actions screened in estrogen-insensitive T47Dco human breast cancer cells. Endocrinology.

[bib84] Horwitz KB (1992). The molecular biology of RU486. Is there a role for antiprogestins in the treatment of breast cancer?. Endocrine Reviews.

[bib85] Huniadi CA, Pop OL, Antal TA, Stamatian F (2013). The effects of ulipristal on Bax/Bcl-2, cytochrome *c*, Ki-67 and cyclooxygenase-2 expression in a rat model with surgically induced endometriosis. European Journal of Obstetrics, Gynecology, and Reproductive Biology.

[bib86] Ibrahim YH, Byron SA, Cui X, Lee AV, Yee D (2008). Progesterone receptor-B regulation of insulin-like growth factor-stimulated cell migration in breast cancer cells via insulin receptor substrate-2. Molecular Cancer Research.

[bib87] Jeng MH, Langan-Fahey SM, Jordan VC (1993). Estrogenic actions of RU486 in hormone-responsive MCF-7 human breast cancer cells. Endocrinology.

[bib88] Jonat W, Bachelot T, Ruhstaller T, Kuss I, Reimann U, Robertson JF (2013). Randomized phase II study of lonaprisan as second-line therapy for progesterone receptor-positive breast cancer. Annals of Oncology.

[bib89] Josefsberg Ben-Yehoshua L, Lewellyn AL, Thomas P, Maller JL (2007). The role of *Xenopus* membrane progesterone receptor β in mediating the effect of progesterone on oocyte maturation. Molecular Endocrinology.

[bib90] Journe F, Body JJ, Leclercq G, Laurent G (2008). Hormone therapy for breast cancer, with an emphasis on the pure antiestrogen fulvestrant: mode of action, antitumor efficacy and effects on bone health. Expert Opinion on Drug Safety.

[bib91] Jurado R, Lopez-Flores A, Alvarez A, Garcia-Lopez P (2009). Cisplatin cytotoxicity is increased by mifepristone in cervical carcinoma: an *in vitro* and *in vivo* study. Oncology Reports.

[bib92] Kamradt MC, Mohideen N, Vaughan AT (2000). RU486 increases radiosensitivity and restores apoptosis through modulation of HPV E6/E7 in dexamethasone-treated cervical carcinoma cells. Gynecologic Oncology.

[bib93] Keith Bechtel M, Bonavida B (2001). Inhibitory effects of 17β-estradiol and progesterone on ovarian carcinoma cell proliferation: a potential role for inducible nitric oxide synthase. Gynecologic Oncology.

[bib94] Kersey JP, Broadway DC (2006). Costicosteroid-induced glaucoma: a review of the literature. Eye.

[bib95] Kettel LM, Murphy AA, Mortola JF, Liu JH, Ulmann A, Yen SS (1991). Endocrine responses to long-term administration of the antiprogesterone RU486 in patients with pelvic endometriosis. Fertility and Sterility.

[bib96] Kettel LM, Murphy AA, Morales AJ, Yen SS (1994). Clinical efficacy of the antiprogesterone RU486 in the treatment of endometriosis and uterine fibroids. Human Reproduction.

[bib97] Kettel LM, Murphy AA, Morales AJ, Ulmann A, Baulieu EE, Yen SS (1996). Treatment of endometriosis with the antiprogesterone mifepristone (RU486). Fertility and Sterility.

[bib98] Klijn JG, de Jong FH, Bakker GH, Lamberts SW, Rodenburg CJ, Alexieva-Figusch J (1989). Antiprogestins, a new form of endocrine therapy for human breast cancer. Cancer Research.

[bib99] Kloosterboer HJ, Deckers GH, Schoonen WG, Hanssen RG, Rose UM, Verbost PM, Hsiu JG, Williams RF, Hodgen GD (2000). Preclinical experience with two selective progesterone receptor modulators on breast and endometrium. Steroids.

[bib215] Knutson TP, Daniel AR, Fan D, Silverstein KA, Covington KR, Fuqua SA, Lange CA (2012). Phosphorylated and sumoylation-deficient progesterone receptors drive proliferative gene signatures during breast cancer progression. Breast Cancer Research.

[bib100] Knutson TP, Lange CA (2014). Tracking progesterone receptor-mediated actions in breast cancer. Pharmacology & Therapeutics.

[bib101] Koivisto-Korander R, Leminen A, Heikinheimo O (2007). Mifepristone as treatment of recurrent progesterone receptor-positive uterine leiomyosarcoma. Obstetrics and Gynecology.

[bib102] Lamb C, Simian M, Molinolo A, Pazos P, Lanari C (1999). Regulation of cell growth of a progestin-dependent murine mammary carcinoma *in vitro*: progesterone receptor involvement in serum or growth factor-induced cell proliferation. Journal of Steroid Biochemistry and Molecular Biology.

[bib103] Lamb CA, Helguero LA, Giulianelli S, Soldati R, Vanzulli SI, Molinolo A, Lanari C (2005). Antisense oligonucleotides targeting the progesterone receptor inhibit hormone-independent breast cancer growth in mice. Breast Cancer Research.

[bib104] Lanari C, Lamb CA, Fabris VT, Helguero LA, Soldati R, Bottino MC, Giulianelli S, Cerliani JP, Wargon V, Molinolo A (2009). The MPA mouse breast cancer model: evidence for a role of progesterone receptors in breast cancer. Endocrine-Related Cancer.

[bib105] Lanari C, Wargon V, Rojas P, Molinolo AA (2012). Antiprogestins in breast cancer treatment: are we ready?. Endocrine-Related Cancer.

[bib106] Lents NH, Keenan SM, Bellone C, Baldassare JJ (2002). Stimulation of the Raf/MEK/ERK cascade is necessary and sufficient for activation and Thr-160 phosphorylation of a nuclear-targeted CDK2. Journal of Biological Chemistry.

[bib107] Lessey BA, Ilesanmi AO, Castelbaum AJ, Yuan L, Somkuti SG, Chwalisz K, Satyaswaroop PG (1996). Characterization of the functional progesterone receptor in an endometrial adenocarcinoma cell line (Ishikawa): progesterone-induced expression of the α1 integrin. Journal of Steroid Biochemistry and Molecular Biology.

[bib108] Levens ED, Potlog-Nahari C, Armstrong AY, Wesley R, Premkumar A, Blithe DL, Blocker W, Nieman LK (2008). CDB-2914 for uterine leiomyomata treatment: a randomized controlled trial. Obstetrics and Gynecology.

[bib109] Lewis-Tuffin LJ, Jewell CM, Bienstock RJ, Collins JB, Cidlowski JA (2007). Human glucocorticoid receptor β binds RU-486 and is transcriptionally active. Molecular and Cellular Biology.

[bib110] Li Q, Li JJ, Zhao XB, Ji M (2003). Study of effect of mifepristone on apoptosis of human ovarian cancer cell line 3AO. Zhonghua fu chan ke za zhi.

[bib111] Li DQ, Wang ZB, Bai J, Zhao J, Wang Y, Hu K, Du YH (2004a). Effects of mifepristone on proliferation of human gastric adenocarcinoma cell line SGC-7901 *in vitro*. World Journal of Gastroenterology.

[bib112] Li DQ, Wang ZB, Bai J, Zhao J, Wang Y, Hu K, Du YH (2004b). Effects of mifepristone on invasive and metastatic potential of human gastric adenocarcinoma cell line MKN-45 *in vitro* and *in vivo*. World Journal of Gastroenterology.

[bib113] Li A, Felix JC, Minoo P, Amezcua CA, Jain JK (2005). Effect of mifepristone on proliferation and apoptosis of Ishikawa endometrial adenocarcinoma cells. Fertility and Sterility.

[bib114] Liang Y, Eid MA, El Etreby F, Lewis RW, Kumar MV (2002). Mifepristone-induced secretion of transforming growth factor β1-induced apoptosis in prostate cancer cells. International Journal of Oncology.

[bib115] Liang Y, Hou M, Kallab AM, Barrett JT, El Etreby F, Schoenlein PV (2003). Induction of antiproliferation and apoptosis in estrogen receptor negative MDA-231 human breast cancer cells by mifepristone and 4-hydroxytamoxifen combination therapy: a role for TGFβ1. International Journal of Oncology.

[bib116] Liberto M, Cobrinik D (2000). Growth factor-dependent induction of p21(CIP1) by the green tea polyphenol, epigallocatechin gallate. Cancer Letters.

[bib117] Lin VC, Aw SE, Ng EH, Ng EH, Tan MG (2001). Demonstration of mixed properties of RU486 in progesterone receptor (PR)-transfected MDA-MB-231 cells: a model for studying the functions of progesterone analogues. British Journal of Cancer.

[bib118] Liu M, Wikonkal NM, Brash DE (1999). Induction of cyclin-dependent kinase inhibitors and G(1) prolongation by the chemopreventive agent *N*-acetylcysteine. Carcinogenesis.

[bib119] Long XE, Gong ZH, Pan L, Zhong ZW, Le YP, Liu Q, Guo JM, Zhong JC (2010). Suppression of CDK2 expression by siRNA induces cell cycle arrest and cell proliferation inhibition in human cancer cells. BMB Reports.

[bib120] Luo X, Yin P, Coon VJ, Cheng YH, Wiehle RD, Bulun SE (2010). The selective progesterone receptor modulator CDB4124 inhibits proliferation and induces apoptosis in uterine leiomyoma cells. Fertility and Sterility.

[bib121] Mao J, Regelson W, Kalimi M (1992). Molecular mechanism of RU 486 action: a review. Molecular and Cellular Biochemistry.

[bib122] Matsuda Y, Kawamoto K, Kiya K, Kurisu K, Sugiyama K, Uozumi T (1994). Antitumor effects of antiprogesterones on human meningioma cells *in vitro* and *in vivo*. Journal of Neurosurgery.

[bib123] McDonnel AC, Murdoch WJ (2001). High-dose progesterone inhibition of urokinase secretion and invasive activity by SKOV-3 ovarian carcinoma cells: evidence for a receptor-independent nongenomic effect on the plasma membrane. Journal of Steroid Biochemistry and Molecular Biology.

[bib124] Mei L, Bao J, Tang L, Zhang C, Wang H, Sun L, Ma G, Huang L, Yang J, Zhang L (2010). A novel mifepristone-loaded implant for long-term treatment of endometriosis: *in vitro* and *in vivo* studies. European Journal of Pharmaceutical Sciences.

[bib125] Meijer CJ, Snijders PJ (2014). Cervical cancer in 2013: screening comes of age and treatment progress continues. Nature Reviews. Clinical Oncology.

[bib126] Michna H, Schneider MR, Nishino Y, el Etreby MF (1989). Antitumor activity of the antiprogestins ZK 98.299 and RU 38.486 in hormone dependent rat and mouse mammary tumours: mechanistic studies. Breast Cancer Research and Treatment.

[bib127] Michna H, Nishino Y, Neef G, McGuire WL, Schneider MR (1992). Progesterone antagonists: tumor-inhibiting potential and mechanism of action. Journal of Steroid Biochemistry and Molecular Biology.

[bib128] Milewicz T, Gregoraszczuk EL, Sztefko K, Augustowska K, Krzysiek J, Rys J (2005). Lack of synergy between estrogen and progesterone on local IGF-I, IGFBP-3 and IGFBP-2 secretion by both hormone-dependent and hormone-independent breast cancer explants *in vitro*. Effect of tamoxifen and mifepristone (RU 486). Growth Hormone & IGF Research.

[bib129] Modugno F, Laskey R, Smith AL, Andersen CL, Haluska P, Oesterreich S (2012). Hormone response in ovarian cancer: time to reconsider as a clinical target?. Endocrine-Related Cancer.

[bib130] Moe BG, Vereide AB, Orbo A, Sager G (2009). High concentrations of progesterone and mifepristone mutually reinforce cell cycle retardation and induction of apoptosis. Anticancer Research.

[bib131] Molenaar JJ, Ebus ME, Geerts D, Koster J, Lamers F, Valentijn LJ, Westerhout EM, Versteeg R, Caron HN (2009). Inactivation of CDK2 is synthetically lethal to MYCN over-expressing cancer cells. PNAS.

[bib132] Montecchia MF, Lamb C, Molinolo AA, Luthy IA, Pazos P, Charreau E, Vanzulli S, Lanari C (1999). Progesterone receptor involvement in independent tumor growth in MPA-induced murine mammary adenocarcinomas. Journal of Steroid Biochemistry and Molecular Biology.

[bib133] Murphy AA, Castellano PZ (1994). RU486: pharmacology and potential use in the treatment of endometriosis and leiomyomata uteri. Current Opinion in Obstetrics & Gynecology.

[bib134] Murphy AA, Kettel LM, Morales AJ, Roberts VJ, Yen SS (1993). Regression of uterine leiomyomata in response to the antiprogesterone RU 486. Journal of Clinical Endocrinology and Metabolism.

[bib135] Murphy AA, Morales AJ, Kettel LM, Yen SS (1995). Regression of uterine leiomyomata to the antiprogesterone RU486: dose–response effect. Fertility and Sterility.

[bib136] Murphy AA, Zhou MH, Malkapuram S, Santanam N, Parthasarathy S, Sidell N (2000). RU486-induced growth inhibition of human endometrial cells. Fertility and Sterility.

[bib137] Musgrove EA, Lee CS, Cornish AL, Swarbrick A, Sutherland RL (1997). Antiprogestin inhibition of cell cycle progression in T-47D breast cancer cells is accompanied by induction of the cyclin-dependent kinase inhibitor p21. Molecular Endocrinology.

[bib138] Muti P (2014). Is progesterone a neutral or protective factor for breast cancer?. Nature Reviews. Cancer.

[bib139] Nardella C, Clohessy JG, Alimonti A, Pandolfi PP (2011). Pro-senescence therapy for cancer treatment. Nature Reviews. Cancer.

[bib140] Nardulli AM, Katzenellenbogen BS (1988). Progesterone receptor regulation in T47D human breast cancer cells: analysis by density labeling of progesterone receptor synthesis and degradation and their modulation by progestin. Endocrinology.

[bib141] Navo MA, Smith JA, Gaikwad A, Burke T, Brown J, Ramondetta LM (2008). *In vitro* evaluation of the growth inhibition and apoptosis effect of mifepristone (RU486) in human Ishikawa and HEC1A endometrial cancer cell lines. Cancer Chemotherapy and Pharmacology.

[bib142] Nickisch K, Nair HB, Kesavaram N, Das B, Garfield R, Shi SQ, Bhaskaran SS, Grimm SL, Edwards DP (2013). Synthesis and antiprogestational properties of novel 17-fluorinated steroids. Steroids.

[bib143] Nieman LK, Chrousos GP, Kellner C, Spitz IM, Nisula BC, Cutler GB, Merriam GR, Bardin CW, Loriaux DL (1985). Successful treatment of Cushing's syndrome with the glucocorticoid antagonist RU 486. Journal of Clinical Endocrinology and Metabolism.

[bib144] Oakley RH, Jewell CM, Yudt MR, Bofetiado DM, Cidlowski JA (1999). The dominant negative activity of the human glucocorticoid receptor β isoform. Specificity and mechanisms of action. Journal of Biological Chemistry.

[bib145] Parthasarathy S, Morales AJ, Murphy AA (1994). Antioxidant: a new role for RU-486 and related compounds. Journal of Clinical Investigation.

[bib146] Peluso JJ, Liu X, Saunders MM, Claffey KP, Phoenix K (2008). Regulation of ovarian cancer cell viability and sensitivity to cisplatin by progesterone receptor membrane component-1. Journal of Clinical Endocrinology and Metabolism.

[bib147] Perez-Mancera PA, Young AR, Narita M (2014). Inside and out: the activities of senescence in cancer. Nature Reviews. Cancer.

[bib148] Periyasamy-Thandavan S, Takhar S, Singer A, Dohn MR, Jackson WH, Welborn AE, LeRoith D, Marrero M, Thangaraju M, Huang S (2012). Insulin-like growth factor 1 attenuates antiestrogen- and antiprogestin-induced apoptosis in ER+ breast cancer cells by MEK1 regulation of the BH3-only pro-apoptotic protein Bim. Breast Cancer Research.

[bib149] Perrault D, Eisenhauer EA, Pritchard KI, Panasci L, Norris B, Vandenberg T, Fisher B (1996). Phase II study of the progesterone antagonist mifepristone in patients with untreated metastatic breast carcinoma: a National Cancer Institute of Canada Clinical Trials Group study. Journal of Clinical Oncology.

[bib150] Peters MG, Vanzulli S, Elizalde PV, Charreau EH, Goin MM (2001). Effects of antiprogestins RU486 and ZK98299 on the expression of cell cycle proteins of a medroxyprogesterone acetate (MPA)-induced murine mammary tumor. Oncology Reports.

[bib151] **Philibert D, Deraedt R & Teutsch G** 1981 RU 38486: a potent antiglucocorticoid *in vivo*, The VIII International Congress of Pharmacology, Tokio, Japan, Abstract 1463.

[bib152] Pierson-Mullany LK, Lange CA (2004). Phosphorylation of progesterone receptor serine 400 mediates ligand-independent transcriptional activity in response to activation of cyclin-dependent protein kinase 2. Molecular and Cellular Biology.

[bib153] Pinski J, Halmos G, Shirahige Y, Wittliff JL, Schally AV (1993). Inhibition of growth of the human malignant glioma cell line (U87MG) by the steroid hormone antagonist RU486. Journal of Clinical Endocrinology and Metabolism.

[bib154] Polo ML, Arnoni MV, Riggio M, Wargon V, Lanari C, Novaro V (2010). Responsiveness to PI3K and MEK inhibitors in breast cancer. Use of a 3D culture system to study pathways related to hormone independence in mice. PLoS ONE.

[bib155] Poole AJ, Li Y, Kim Y, Lin SC, Lee WH, Lee EY (2006). Prevention of Brca1-mediated mammary tumorigenesis in mice by a progesterone antagonist. Science.

[bib156] Qin TN, Wang LL (2002). Enhanced sensitivity of ovarian cell line to cisplatin induced by mifepristone and its mechanism. Di Yi Jun Yi Da Xue Xue Bao.

[bib157] Ray NC, Clark RD, Clark DE, Williams K, Hickin HG, Crackett PH, Dyke HJ, Lockey PM, Wong M, Devos R (2007). Discovery and optimization of novel, non-steroidal glucocorticoid receptor modulators. Bioorganic & Medicinal Chemistry Letters.

[bib158] Roberts CP, Parthasarathy S, Gulati R, Horowitz I, Murphy AA (1995). Effect of RU-486 and related compounds on the proliferation of cultured macrophages. American Journal of Reproductive Immunology.

[bib159] Robertson JF, Willsher PC, Winterbottom L, Blamey RW, Thorpe S (1999). Onapristone, a progesterone receptor antagonist, as first-line therapy in primary breast cancer. European Journal of Cancer.

[bib160] Rocereto TF, Saul HM, Aikins JA, Paulson J (2000). Phase II study of mifepristone (RU486) in refractory ovarian cancer. Gynecologic Oncology.

[bib161] Rocereto TF, Brady WE, Shahin MS, Hoffman JS, Small L, Rotmensch J, Mannel RS (2010). A phase II evaluation of mifepristone in the treatment of recurrent or persistent epithelial ovarian, fallopian or primary peritoneal cancer: a gynecologic oncology group study. Gynecologic Oncology.

[bib162] Romero I, Bast RC (2012). Minireview: Human ovarian cancer: biology, current management, and paths to personalizing therapy. Endocrinology.

[bib163] Romieu G, Maudelonde T, Ulmann A, Pujol H, Grenier J, Cavalie G, Khalaf S, Rochefort H (1987). The antiprogestin RU486 in advanced breast cancer: preliminary clinical trial. Bulletin du Cancer.

[bib164] Rose FV, Barnea ER (1996). Response of human ovarian carcinoma cell lines to antiprogestin mifepristone. Oncogene.

[bib165] Sadler SE, Bower MA, Maller JL (1985). Studies of a plasma membrane steroid receptor in *Xenopus* oocytes using the synthetic progestin RU 486. Journal of Steroid Biochemistry.

[bib166] Sartorius CA, Groshong SD, Miller LA, Powell RL, Tung L, Takimoto GS, Horwitz KB (1994). New T47D breast cancer cell lines for the independent study of progesterone B- and A-receptors: only antiprogestin-occupied B-receptors are switched to transcriptional agonists by cAMP. Cancer Research.

[bib167] Schneider CC, Gibb RK, Taylor DD, Wan T, Gercel-Taylor C (1998). Inhibition of endometrial cancer cell lines by mifepristone (RU 486). Journal of the Society for Gynecologic Investigation.

[bib168] Schoenlein PV, Hou M, Samaddar JS, Gaddy VT, Thangaraju M, Lewis J, Johnson M, Ganapathy V, Kallab A, Barrett JT (2007). Downregulation of retinoblastoma protein is involved in the enhanced cytotoxicity of 4-hydroxytamoxifen plus mifepristone combination therapy versus antiestrogen monotherapy of human breast cancer. International Journal of Oncology.

[bib169] Schonthal AH (2013). Pharmacological targeting of endoplasmic reticulum stress signaling in cancer. Biochemical Pharmacology.

[bib170] Sequeira G, Vanzulli SI, Rojas P, Lamb C, Colombo L, May M, Molinolo A, Lanari C (2014). The effectiveness of nano chemotherapeutic particles combined with mifepristone depends on the PR isoform ratio in preclinical models of breast cancer. Oncotarget.

[bib171] Sharma R, Yang Y, Sharma A, Awasthi S, Awasthi YC (2004). Antioxidant role of glutathione *S*-transferases: protection against oxidant toxicity and regulation of stress-mediated apoptosis. Antioxidants & Redox Signaling.

[bib172] Shen Q, Hua Y, Jiang W, Zhang W, Chen M, Zhu X (2013). Effects of mifepristone on uterine leiomyoma in premenopausal women: a meta-analysis. Fertility and Sterility.

[bib173] Shi YE, Liu YE, Lippman ME, Dickson RB (1994). Progestins and antiprogestins in mammary tumour growth and metastasis. Human Reproduction.

[bib174] Shimomura Y, Matsuo H, Samoto T, Maruo T (1998). Up-regulation by progesterone of proliferating cell nuclear antigen and epidermal growth factor expression in human uterine leiomyoma. Journal of Clinical Endocrinology and Metabolism.

[bib175] Siegel R, Naishadham D, Jemal A (2013). Cancer statistics, 2013. CA: A Cancer Journal for Clinicians.

[bib176] Sieh W, Kobel M, Longacre TA, Bowtell DD, deFazio A, Goodman MT, Hogdall E, Deen S, Wentzensen N, Moysich KB (2013). Hormone-receptor expression and ovarian cancer survival: an Ovarian Tumor Tissue Analysis consortium study. Lancet. Oncology.

[bib177] Simian M, Molinolo A, Lanari C (2006). Involvement of matrix metalloproteinase activity in hormone-induced mammary tumor regression. American Journal of Pathology.

[bib178] Skor MN, Wonder EL, Kocherginsky M, Goyai A, Hall BA, Cai Y, Conzen SD (2013). Glucocorticoid receptor antagonism as a novel therapy for triple-negative breast cancer. Clinical Cancer Research.

[bib179] Smith JL, Kupchak BR, Garitaonandia I, Hoang LK, Maina AS, Regalla LM, Lyons TJ (2008). Heterologous expression of human mPRα, mPRβ and mPRγ in yeast confirms their ability to function as membrane progesterone receptors. Steroids.

[bib180] Song LN, Coghlan M, Gelmann EP (2004). Antiandrogen effects of mifepristone on coactivator and corepressor interactions with the androgen receptor. Molecular Endocrinology.

[bib181] Spitz IM (2006). Progesterone receptor antagonists. Current Opinion in Investigational Drugs.

[bib182] Spitz IM, Bardin CW (1993). Mifepristone (RU 486) – a modulator of progestin and glucocorticoid action. New England Journal of Medicine.

[bib183] Spitz IM, Croxatto HB, Robbins A (1996). Antiprogestins: mechanism of action and contraceptive potential. Annual Review of Pharmacology and Toxicology.

[bib184] Stoeckemann K, Hegele-Hartung C, Chwalisz K (1995). Effects of the progesterone antagonists onapristone (ZK 98 299) and ZK 136 799 on surgically induced endometriosis in intact rats. Human Reproduction.

[bib185] Sui L, Dong Y, Ohno M, Sugimoto K, Tai Y, Hando T, Tokuda M (2001). Implication of malignancy and prognosis of p27(kip1), cyclin E, and Cdk2 expression in epithelial ovarian tumors. Gynecologic Oncology.

[bib186] Sun QL, Zhang XG, Xing QT, Ding P, Feng JB, Wu XP, Wang ZM (2012). A study of mifepristone/IFN-γ-induced apoptosis of human cholangiocarcinoma cell line FRH-0201 *in vitro*. OncoTargets and Therapy.

[bib187] Tamm-Rosenstein K, Simm J, Suhorutshenko M, Salumets A, Metsis M (2013). Changes in the transcriptome of the human endometrial Ishikawa cancer cell line induced by estrogen, progesterone, tamoxifen, and mifepristone (RU486) as detected by RNA-sequencing. PLoS ONE.

[bib188] Taniguchi Y, Iwasaki Y, Tsugita M, Nishiyama M, Taguchi T, Okazaki M, Nakayama S, Kambayashi M, Hashimoto K, Terada Y (2010). Glucocorticoid receptor-β and receptor-γ exert dominant negative effect on gene repression but not on gene induction. Endocrinology.

[bib189] Telleria CM (2013). Repopulation of ovarian cancer cells after chemotherapy. Cancer Growth and Metastasis.

[bib190] Telleria CM, Goyeneche AA, Farghaly S (2012). Antiprogestins in ovarian cancer. Ovarian Cancer – Clinical and Therapeutic Perspectives.

[bib191] Terakawa N, Shimizu I, Tanizawa O, Matsumoto K (1988). RU486, a progestin antagonist, binds to progesterone receptors in a human endometrial cancer cell line and reverses the growth inhibition by progestins. Journal of Steroid Biochemistry.

[bib192] Thomas P, Pang Y, Dong J, Groenen P, Kelder J, de Vlieg J, Zhu Y, Tubbs C (2007). Steroid and G protein binding characteristics of the seatrout and human progestin membrane receptor α subtypes and their evolutionary origins. Endocrinology.

[bib193] Tieszen CR, Goyeneche AA, Brandhagen BN, Ortbahn CT, Telleria CM (2011). Antiprogestin mifepristone inhibits the growth of cancer cells of reproductive and non-reproductive origin regardless of progesterone receptor expression. BMC Cancer.

[bib194] Tjaden B, Galetto D, Woodruff JD, Rock JA (1993). Time-related effects of RU486 treatment in experimentally induced endometriosis in the rat. Fertility and Sterility.

[bib195] Trevino LS, Weigel NL (2013). Phosphorylation: a fundamental regulator of steroid receptor action. Trends in Endocrinology and Metabolism.

[bib196] Urra H, Dufey E, Lisbona F, Rojas-Rivera D, Hetz C (2013). When ER stress reaches a dead end. Biochimica et Biophysica Acta.

[bib197] Vanzulli S, Efeyan A, Benavides F, Helguero LA, Peters G, Shen J, Conti CJ, Lanari C, Molinolo A (2002). p21, p27 and p53 in estrogen and antiprogestin-induced tumor regression of experimental mouse mammary ductal carcinomas. Carcinogenesis.

[bib198] Vanzulli SI, Soldati R, Meiss R, Colombo L, Molinolo AA, Lanari C (2005). Estrogen or antiprogestin treatment induces complete regression of pulmonary and axillary metastases in an experimental model of breast cancer progression. Carcinogenesis.

[bib199] Vaughan S, Coward JI, Bast RC, Berchuck A, Berek JS, Brenton JD, Coukos G, Crum CC, Drapkin R, Etemadmoghadam D (2011). Rethinking ovarian cancer: recommendations for improving outcomes. Nature Reviews. Cancer.

[bib200] Veeck J, Dahl E (2012). Targeting the Wnt pathway in cancer: the emerging role of Dickkopf-3. Biochimica et Biophysica Acta.

[bib201] Wang J, Chen J, Wan L, Shao J, Lu Y, Zhu Y, Ou M, Yu S, Chen H, Jia L (2014). Synthesis, spectral characterization, and *in vitro* cellular activities of metapristone, a potential cancer metastatic chemopreventive agent derived from mifepristone (RU486). AAPS Journal.

[bib202] Wargon V, Helguero LA, Bolado J, Rojas P, Novaro V, Molinolo A, Lanari C (2009). Reversal of antiprogestin resistance and progesterone receptor isoform ratio in acquired resistant mammary carcinomas. Breast Cancer Research and Treatment.

[bib203] Wempe SL, Gamarra-Luques CD, Telleria CM (2013). Synergistic lethality of mifepristone and LY294002 in ovarian cancer cells. Cancer Growth and Metastasis.

[bib204] Wiehle RD, Christov K, Mehta R (2007). Anti-progestins suppress the growth of established tumors induced by 7,12-dimethylbenz(a)anthracene: comparison between RU486 and a new 21-substituted-19-nor-progestin. Oncology Reports.

[bib205] Xu Q, Takekida S, Ohara N, Chen W, Sitruk-Ware R, Johansson ED, Maruo T (2005). Progesterone receptor modulator CDB-2914 down-regulates proliferative cell nuclear antigen and Bcl-2 protein expression and up-regulates caspase-3 and poly(adenosine 5′-diphosphate-ribose) polymerase expression in cultured human uterine leiomyoma cells. Journal of Clinical Endocrinology and Metabolism.

[bib206] Xu Q, Ohara N, Chen W, Liu J, Sasaki H, Morikawa A, Sitruk-Ware R, Johansson ED, Maruo T (2006). Progesterone receptor modulator CDB-2914 down-regulates vascular endothelial growth factor, adrenomedullin and their receptors and modulates progesterone receptor content in cultured human uterine leiomyoma cells. Human Reproduction.

[bib207] Xu Q, Ohara N, Liu J, Nakabayashi K, DeManno D, Chwalisz K, Yoshida S, Maruo T (2007). Selective progesterone receptor modulator asoprisnil induces endoplasmic reticulum stress in cultured human uterine leiomyoma cells. American Journal of Physiology. Endocrinology and Metabolism.

[bib208] Yerushalmi GM, Gilboa Y, Jakobson-Setton A, Tadir Y, Goldchmit C, Katz D, Seidman DS (2014). Vaginal mifepristone for the treatment of symptomatic uterine leiomyomata: an open-label study. Fertility and Sterility.

[bib209] Yokoyama Y, Shinohara A, Takahashi Y, Wan X, Takahashi S, Niwa K, Tamaya T (2000). Synergistic effects of danazol and mifepristone on the cytotoxicity of UCN-01 in hormone-responsive breast cancer cells. Anticancer Research.

[bib210] Yoshida S, Ohara N, Xu Q, Chen W, Wang J, Nakabayashi K, Sasaki H, Morikawa A, Maruo T (2010). Cell-type specific actions of progesterone receptor modulators in the regulation of uterine leiomyoma growth. Seminars in Reproductive Medicine.

[bib211] Yudt MR, Jewell CM, Bienstock RJ, Cidlowski JA (2003). Molecular origins for the dominant negative function of human glucocorticoid receptor β. Molecular and Cellular Biology.

[bib212] Zhang S, Jonklaas J, Danielsen M (2007). The glucocorticoid agonist activities of mifepristone (RU486) and progesterone are dependent on glucocorticoid receptor levels but not on EC50 values. Steroids.

[bib213] Zhao J, Kennedy BK, Lawrence BD, Barbie DA, Matera AG, Fletcher JA, Harlow E (2000). NPAT links cyclin E-Cdk2 to the regulation of replication-dependent histone gene transcription. Genes and Development.

[bib214] Zhou H, Luo MP, Schonthal AH, Pike MC, Stallcup MR, Blumenthal M, Zheng W, Dubeau L (2002). Effect of reproductive hormones on ovarian epithelial tumors: I. Effect on cell cycle activity. Cancer Biology & Therapy.

