# Supplementary material for: Antiprogestins in gynecological diseases
Source: Reproduction. 2014 Sep 4;149(1):R15–33. doi: 10.1530/REP-14-0416 (PMC4247796; doi:10.1530/REP-14-0416)
Supplement: http://www.reproduction-online.org/content/149/1/R15/suppl/DC1 [file supp_149_1_R15__index.html]

Free Spanish Abstract 

# Antiprogestins in gynecological diseases

## Free Spanish Abstract

A Spanish translation of the abstract is freely available here. (PDF 186Kb)

**Files in this Data Supplement:**

- Adobe PDF - Spanish\_abstract\_REP-14-0416.pdf
