# Supplementary material for: Antiprogestins in gynecological diseases
Source: Reproduction. 2014 Sep 4;149(1):R15–33. doi: 10.1530/REP-14-0416 (PMC4247796; doi:10.1530/REP-14-0416)
Supplement: http://www.reproduction-online.org/content/149/1/R15/suppl/DC1 [file supp_REP-14-0416_Spanish_abstract_REP-14-0416.pdf]

Antiprogestinas en enfermedades ginecológicas

Las antiprogestinas representan un grupo de esteroides que comenzaron a sintetizarse a principios de la década de los ´80s. Estos compuestos se unen a los receptores de progesterona con diferentes afinidades. Los primeros usos clínicos de las antiprogestinas fueron en el campo de la medicina reproductiva, con el fin de regular la menstruación, como método anticonceptivo de emergencia, o para terminar gestaciones tempranas. Estos usos médicos iniciales, sin embargo, obstaculizaron la utilización de estos compuestos en situaciones médicas en las cuales se necesita interrumpir el crecimiento celular. Dentro del contexto de las enfermedades ginecológicas, las antiprogestinas son capaces de inhibir el crecimiento y de inducir la muerte de células cancerígenas, tales como las que se originan en los cánceres de mama, ovario, endometrio, y cuello de útero. Las antiprogestinas son también capaces de interumpir el crecimiento excesivo de células que causan transtornos ginecológicos benignos, como lo son la endometriosis y los fibroides uterinos. En este artículo, presentamos una revisión de la literatura sobre los efectos de las antiprogestinas inhibiendo el crecimiento de células que representan varias enfermedades ginecológicas. También brindamos un resúmen de los mecanismos moleculares descritos en la literatura científica para estos compuestos químicos y que conllevan a la inhibición del crecimiento y la muerte celular. Los estudios preclínicos realizados hasta el presente proveen evidencias suficientes para anticipar el uso de las antiprogestinas, en un futuro cercano, para aliviar el sufrimiento causado por el excesivo crecimiento celular que ocurre durante el progreso de ciertas enfermedades ginecológicas. Estas antiprogestinas podrían utilizarse como monoterapias o como parte de terapias combinadas con otras medicaciones con el fin de crear tratamientos de larga duración que no causen efectos secundarios indeseados. La clave del éxito clínico del uso de las antiprogestinas para tratar las enfermedades ginecológicas asociadas con el exceso de crecimiento celular será, probablemente, la selección apropiada de los pacientes que tengan las mayores probabilidades de beneficiarse con el tratamiento. Esto podría lograrse si se determina el perfil genético que los pacientes deben poseer para responder positivamente a la terapia de inhibición de crecimiento celular con antiprogestinas.

Palabras claves:
Antiprogestinas - Moduladores de los receptores de progesterona - Tumores del tracto genital femenino - Cánceres ginecológicos - Inhibición del crecimiento celular.
